# Selenium Nanobiostimulants Attenuate Copper-Induced Oxidative Damage in *Brassica napus* Through Genotype-Specific Antioxidant and Metabolic Adaptation

**DOI:** 10.3390/plants15091333

**Published:** 2026-04-27

**Authors:** Sundas Fatima, Muhammad Arslan Yousaf, Saba Yaseen, Muhammad Kamran, Basharat Ali, Yingying Zhou, Asad Ullah, Fangbin Cao, Skhawat Ali, Weijun Zhou

**Affiliations:** 1 Zhejiang Key Laboratory of Crop Germplasm Innovation and Utilization, Institute of Crop Science, Zhejiang University, Hangzhou 310058, China; 2 Department of Agricultural Engineering, Khwaja Fareed University of Engineering and Information Technology, Rahim Yar Khan 64200, Pakistan

**Keywords:** selenium nanoparticles, copper toxicity, *Brassica napus*, phytoremediation, oxidative stress, antioxidant enzymes, secondary metabolites, cultivar-specific responses

## Abstract

Copper (Cu) contamination poses severe threats to agricultural productivity and food safety, particularly affecting economically important crops such as rapeseed (*Brassica napus* L.). This study investigated the protective effects of selenium nanoparticles (SeNPs) against Cu toxicity in four *B. napus* cultivars. Exposure to Cu (200 μM) caused severe reductions in growth and photosynthetic efficiency while significantly elevating oxidative stress markers across all cultivars. Application of SeNPs (25 μM) effectively mitigated these adverse effects, improving biomass, restoring chlorophyll content, and enhancing photosynthetic performance compared to Cu-stressed plants. SeNP treatment significantly enhanced antioxidant enzyme activities, with corresponding upregulation of antioxidant gene expression. Secondary metabolite profiling revealed cultivar-specific responses, with sensitive cultivar Zheda 622 exhibiting metabolic adaptation and higher volatile organic compound (VOC) accumulation, while tolerant cultivar Zheda 635 maintained metabolic stability. PCA analysis demonstrated distinct metabolic clustering patterns, reflecting differential stress-responsive strategies. The study demonstrates that SeNPs attenuate Cu-induced toxicity through integrated mechanisms encompassing diminished Cu acquisition, augmented antioxidant defense systems, and comprehensive metabolic reprogramming. Cultivar-specific responses highlighted substantial genetic variation in tolerance mechanisms across *B. napus* genotypes. These findings substantiate SeNPs as a viable and efficacious nanomaterial for sustainable agronomic management in Cu-contaminated edaphic environments. The approach offers dual benefits of improved crop productivity and reduced Cu accumulation, ensuring enhanced food safety.

## 1. Introduction

*Brassica napus* L., commonly known as rapeseed (canola), represents one of the most economically significant oilseed crops cultivated globally for edible oil production and, increasingly, as a renewable biofuel source [1,2]. Globally, *B. napus* is cultivated across approximately 36.4 million hectares, yielding around 72.5 million tons annually, rendering it one of the world’s most economically significant oilseed crops [3]. Beyond its nutritional and economic value, *B. napus* exhibits several advantageous agronomic traits, including rapid growth rate, high shoot biomass, and adaptability to diverse edaphic conditions [4]. The *Brassicaceae* family has a notable capacity for toxic heavy metal (HMs) ion accumulation, rendering it a promising candidate for phytoremediation applications [5]. Additionally, its responsiveness to HM stress makes it a suitable model for evaluating mitigation strategies. HM contamination, primarily attributed to inadequate industrial waste management practices, constitutes a significant environmental and public health concern.

Copper (Cu), cadmium (Cd), chromium (Cr), lead (Pb), and arsenic (As) are major environmental contaminants due to their toxicity and ability to accumulate in the environment [6]. Of these contaminants, Cu is selected in the present study due to its widespread occurrence in agricultural soils and its dual role as an essential micronutrient integral to key biochemical processes, including cellular respiration, enzyme activation, and photosynthetic electron transport. However, at elevated concentrations, Cu becomes highly phytotoxic, inducing oxidative damage and posing a significant threat to plant growth and productivity [7,8]. Elevated Cu accumulation potentiates excessive reactive oxygen species (ROS) generation, thereby perturbing cellular redox homeostasis and culminating in oxidative stress-mediated cellular damage. This oxidative stress suppresses antioxidant enzyme activity, reduces chlorophyll content, and impairs photosynthetic efficiency, ultimately limiting biomass accumulation and crop productivity [9]. Furthermore, elevated Cu concentrations adversely affect root morphology and nutrient acquisition, exacerbating detrimental effects on plant growth and yield [8,10]. Therefore, effective strategies are required to mitigate Cu-induced phytotoxicity and sustain crop productivity.

To address these challenges, nanotechnology has emerged as a pivotal agricultural strategy, wherein engineered nanoparticles (<100 nm) exploit their ultrafine dimensions to traverse cellular membranes, thereby augmenting crop productivity, fortifying abiotic stress tolerance, and optimizing nutrient use efficiency [11]. Among various nanoparticles, selenium nanoparticles (SeNPs) have received considerable scientific attention due to their remarkable capacity to ameliorate both biotic and abiotic stress conditions [12,13,14]. Compared to conventional Selenium (Se) formulations, SeNPs exhibit superior efficacy in delivering nutritional support and antioxidant protection while promoting enhanced Se acquisition by plants [15,16]. SeNPs demonstrate potential in alleviating diverse environmental stresses, including drought, HMs toxicity, salinity, heat stress, and pathogenic infections [13]. Mechanistically, SeNPs exhibit substantial adsorption capacity for HMs, rendering them effective agents for the remediation of contaminated soil and water [17,18]. Additionally, SeNPs exert pronounced antioxidant effects, stimulate root development and organogenesis, and reinforce plant immunological responses [19]. For instance, ref. [20] demonstrated that soil-applied SeNPs reduced Cd accumulation in bok choy (*Brassica rapa* subsp. *chinensis*) while decreasing Cd absorption rates. Studies on *Coriandrum sativum* cultivars demonstrated that seed priming with SeNP application induced Cd stress tolerance through enhanced antioxidant enzyme activities and increased chlorophyll and total soluble sugar content [21]. Furthermore, similar beneficial effects were observed in barley with improved crop efficiency by reducing Pb bioavailability and increasing plant resistance to oxidative stress [22]. In wheat, SeNPs effectively alleviated Cr toxicity by increasing net photosynthesis rate (Pn) and stomatal conductance (gs), reducing MDA and H_2_O_2_ while enhancing antioxidant enzyme activities [23]. Across these diverse crops and metal stressors, SeNPs promote sustainable agricultural practices by reducing dependence on chemical pesticides and fertilizers while improving crop quality and productivity [24]. However, despite these advances, the mechanisms underlying SeNP-mediated mitigation of Cu toxicity, particularly in relation to cultivar-specific physiological, biochemical, and metabolic responses in *B. napus*, remain insufficiently explored.

Therefore, this study aims to investigate the role of SeNPs in mitigating Cu-induced toxicity in *B. napus* by evaluating growth, physiological, biochemical, and molecular responses across different cultivars. We hypothesize that SeNPs enhance Cu tolerance by reducing oxidative stress, improving antioxidants defense system, and modulating metabolic responses in a cultivar-dependent manner. Unlike previous studies, which focused on a single genotype or limited parameters, the present study simultaneously evaluates multiple *B. napus* cultivars with contrasting Cu tolerance levels. The novelty of this study lies in the integrated assessment of cultivar-specific responses, including metabolic profiling and gene expression analysis, to elucidate the mechanisms of SeNP-mediated Cu stress mitigation in *B. napus*.

## 2. Results

### 2.1. Structural and Morphological Characterization of SeNPs

**Figure 1:**
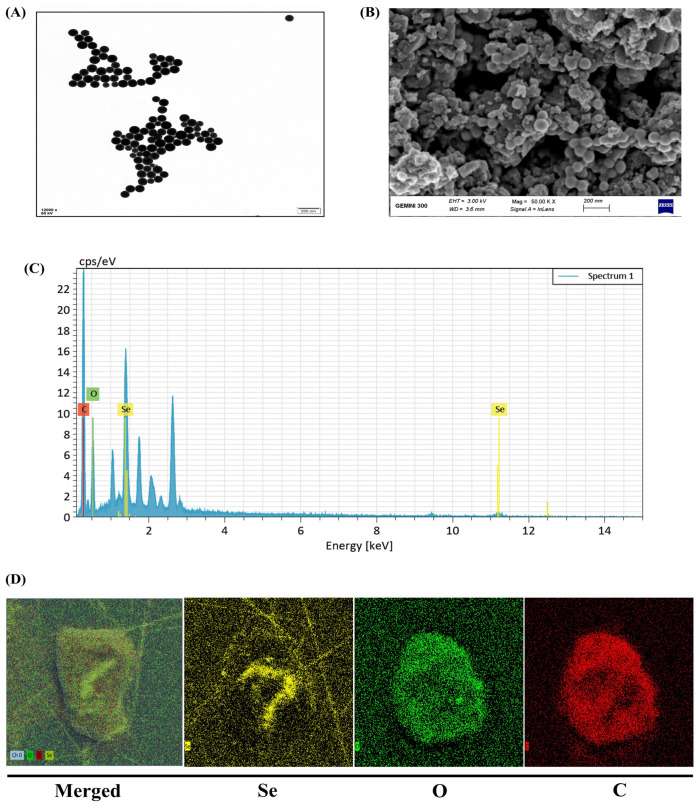
Physicochemical characterization of selenium nanoparticles (SeNPs) showing (**A**) TEM micrograph (scale bar = 200 nm), (**B**) SEM micrograph (scale bar = 200 nm), (**C**) EDX spectrum, and (**D**) elemental composition.

The morphological, structural, and elemental characterization of SeNPs is shown in Figure 1. Transmission electron microscopy (TEM) analysis revealed that SeNPs predominantly possess spherical morphology and clustered aggregates, demonstrating SeNPs’ formation in good uniformity (Figure 1A). Scanning electron microscopy (SEM) was used to assess particle size distribution, confirming the agglomerated granular morphology of SeNPs with particle sizes ranging from 40 to 60 nm (Figure 1B). The elemental composition of SeNPs was evaluated through SEM coupled with EDX (Figure 1C), which confirms the presence of Se as a major element, with C and O confirming the chemical composition of the SeNPs. Furthermore, energy-dispersive X-ray spectroscopy (EDS) elemental mapping identified the homogenous spatial Se in the NP structure, while O and C support effective surface functionalization (Figure 1D).

Additionally, Fourier-transform infrared (FTIR) analysis identified multiple absorption bands corresponding to functional groups such as O-H, C-H, C-O, and C-N, with prominent peaks detected at 3435.56, 2870.04, 1657.03, 1458.40, 1100.67, 702.92, and 463.79 cm^−1^ (Figure S2A). The X-ray diffraction (XRD) analysis confirmed the crystalline nature of SeNPs (Figure S2B). Distinct diffraction peaks ascribed to the trigonal phases of Se confirm the crystalline nature of the NP structure.

### 2.2. Impact of Exogenous SeNPs and Cu on Plant Development and Biomass Accumulation

To evaluate Cu-induced phytotoxicity in *B. napus* cultivars, plant morphological parameters, including fresh and dry biomass, root length, and shoot length, were measured under varying Cu concentrations (0, 100, and 200 μM). Under control conditions, no significant cultivar differences in growth were detected among all cultivars, while exposure to a Cu concentration (100 μM) resulted in significant but comparatively less severe toxic effects than those observed at 200 μM Cu (Figure S1A,B). In contrast, a higher Cu concentration (200 μM) caused pronounced inhibition in leaf fresh weight (LFW), leaf dry weight (LDW), root fresh weight (RFW), root dry weight (RDW), shoot length (SL), and root length (RL) in cultivars Zheda 635, Zheda 630, ZS 758, and Zheda 622 relative to their respective controls (Figure S3A–F). All growth parameters, including LFW, LDW, RFW, RDW, SL, and RL, showed significant reductions under 200 μM Cu stress across all cultivars, with Zheda 622 showing the greatest decline and Zheda 635 the least. Overall, under 200 μM Cu stress, Zheda 622 (most sensitive) showed the greatest reduction in plant biomass/growth, while Zheda 635 (most tolerant) experienced the least reduction, which suggests its relatively better tolerance to Cu stress. Cu-induced biomass reduction likely results from disruption of essential nutrient uptake, impairment of photosynthetic machinery, and excessive ROS accumulation, which collectively compromise cell division and elongation in both root and shoot tissues.

The application of SeNPs (25 μM) significantly alleviated the deleterious effects of Cu stress across *B. napus* cultivars (Zheda 635, Zheda 630, ZS 758, and Zheda 622) (Figure S3). LFW increased by 24.32%, 23.61%, 21.20%, and 20.87%, respectively, relative to plants exposed to Cu (200 μM). Correspondingly, LDW increased by 25.99%, 22.49%, 25.30%, and 20.80% in the same cultivars. RFW exhibited increases of 24.04%, 20.63%, 23.46%, and 14.65%, while RDW improved by 21.21%, 14.86%, 13.99%, and 10.06%, respectively. In addition, SL recovered, increasing by 18%, 15.92%, 12.22%, and 9.92%, whereas root length increased by 17.02%, 33.88%, 21.93%, and 22.72% across the respective cultivars (Figure S3). Notably, the maximum increase in plant biomass was observed in Zheda 635, while the minimum improvement was recorded in Zheda 622, indicating that Zheda 635 was the most tolerant and Zheda 622 was the most sensitive cultivar to Cu stress.

### 2.3. Impact of Exogenous SeNPs and Cu on Photosynthetic Performance

**Figure 2:**
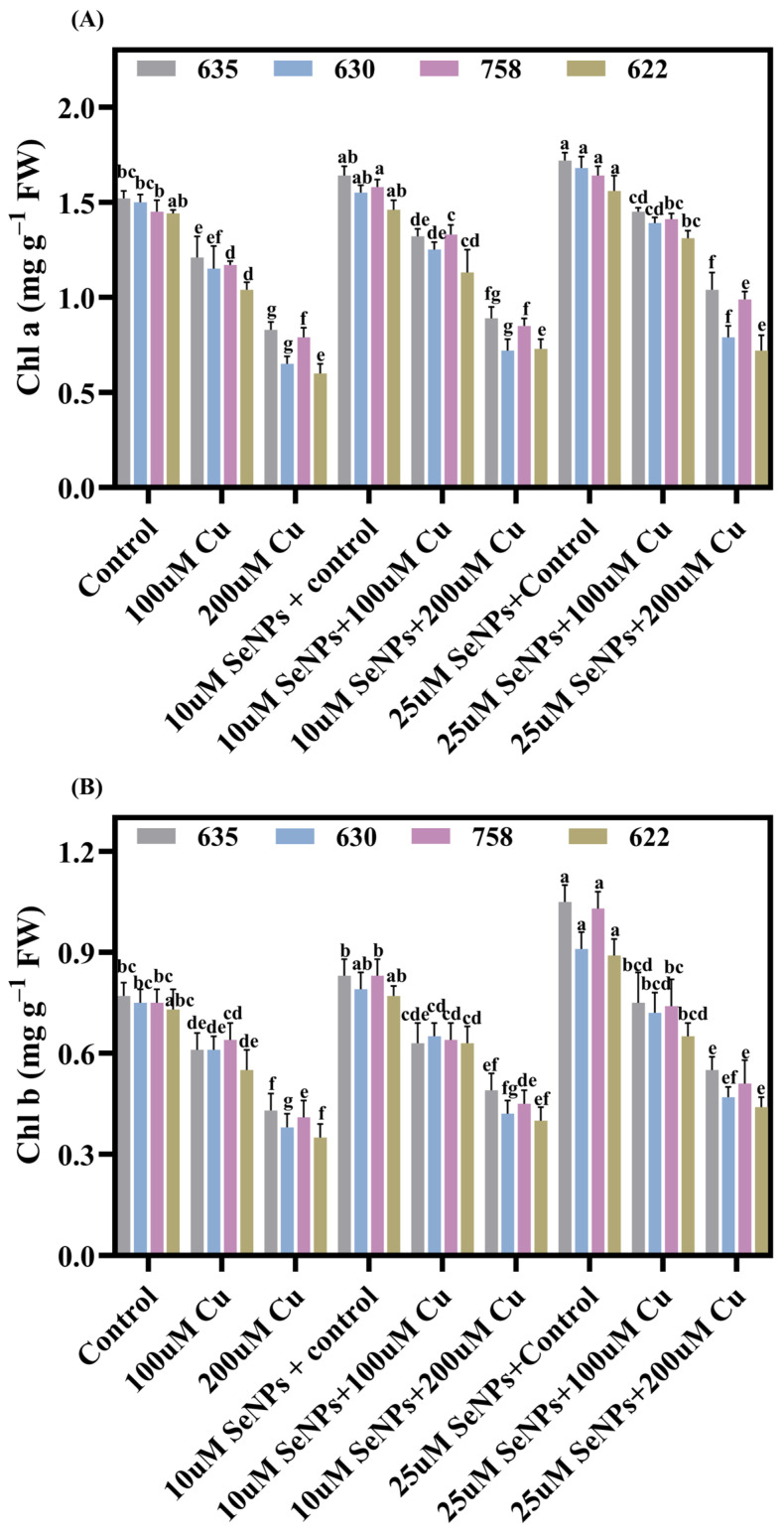

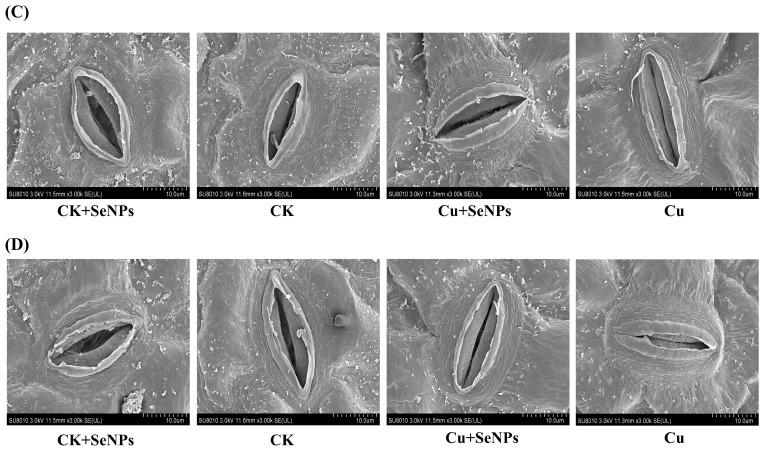
Synergistic effects of copper (0, 100, and 200 μM) and exogenous SeNPs (10 and 25 μM) and their interactions on chlorophyll content in leaves of *B. napus* cultivars (Zheda 635, Zheda 630, ZS 758, and Zheda 622): (**A**) Chlorophyll a (Chl a), (**B**) Chlorophyll b (Chl b), and stomatal opening in Zheda 635 (most tolerant) and Zheda 622 (most sensitive) (**C**,**D**). Bars are presented as mean ± SD (*n* = 3). Bars sharing the same lowercase letter are not significantly different, whereas bars with different letters differ significantly according to Tukey’s HSD test at *p* ≤ 0.05, with comparisons made within each cultivar.

Data on chlorophyll a (Chl a) and chlorophyll b (Chl b) contents in the leaves of *B. napus* varied significantly among all cultivars across the treatments, as presented in Figure 2A,B. Exposure to Cu at 200 µM caused a significant reduction in Chl contents in *B. napus* compared with control treatments. Exposure to 200 μM Cu led to marked decreases in both Chl a and Chl b levels in all cultivars, with the most pronounced reduction observed in Zheda 622 and the smallest change in Zheda 635. (Figure 2A,B). Among the cultivars, Zheda 622 exhibited the greatest susceptibility to Cu-induced inhibition. In contrast, SeNPs (25µM) marginally enhanced Chl a and Chl b in all cultivars relative to their respective controls. The application of SeNPs (25 µM) resulted in a significant enhancement of Chl a by 25.40%, 22.05%, 25.21%, and 21.23%, and Chl b by 27.91%, 24.56%, 25.20%, and 24.53%, respectively, compared with 200 µM Cu concentrations (Figure 2A,B). 

**Figure 3:**
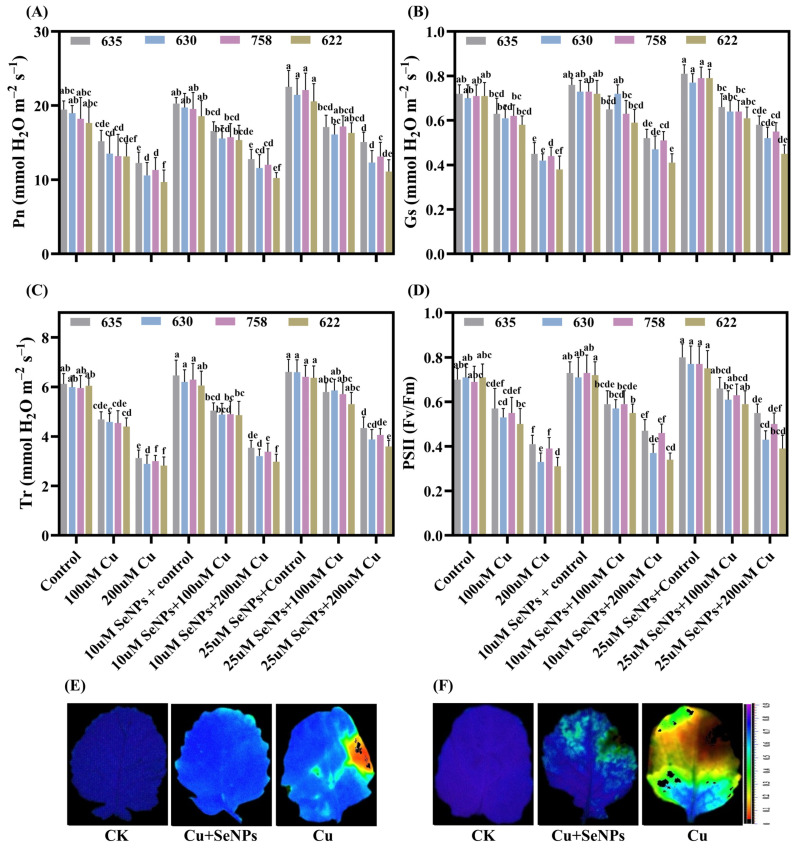
Synergistic effects of copper (0, 100 and 200 μM) and exogenous SeNPs (10 and 25 μM) and their interactions on photosynthesis related traits in leaves of *B. napus* cultivars (Zheda 635, Zheda 630, ZS 758, and Zheda 622): (**A**) net photosynthetic rate (Pn), (**B**) stomatal conductance (Gs), (**C**) transpiration rate (Tr), and (**D**) photochemical efficiency PSII (Fv/Fm) and its visual representation in Zheda 635 (most tolerant) and Zheda 622 (most sensitive) (**E**,**F**). Bars are presented as mean ± SD (*n* = 3). Bars sharing the same lowercase letter are not significantly different, whereas bars with different letters differ significantly according to Tukey’s HSD test at *p* ≤ 0.05, with comparisons made within each cultivar.

The standalone application of SeNPs resulted in modest improvements in gas exchange (IRGA) attributes, encompassing net photosynthetic rate (Pn), stomatal conductance (gs), and transpiration rate (Tr) alongside the efficiency of photosystem II (PSII), relative to untreated control groups (Figure 3A–D). Copper at 200 μM substantially reduced Pn, gs, Tr, and Fv/Fm across all cultivars, with Zheda 622 showing the maximum decrease and Zheda 635 the minimum reduction, respectively, compared to corresponding control plants (Figure 3A–D). Notably, Zheda 622 exhibited the maximum decrease, whereas Zheda 635 demonstrated the minimum reduction under Cu stress conditions across all gas exchange parameters. The application of SeNPs (25µM) markedly enhanced photosynthetic characteristics, demonstrating increases in Pn (by 23.11%, 16.53%, 16.02%, and 14.72%), gs (by 28.89%, 24.60%, 25.95%, and 20.35%), Tr (38.81%, 34.37%, 35.60%, and 27.39%), and PSII efficiency (by 34.15%, 31.63%, 29.06%, and 24.73%), respectively, when compared with Cu (200 µM) treatments (Figure 3A–D).

Microscopic analysis (SEM) was conducted on the epidermal tissue of two *B. napus* cultivars (most tolerant: Zheda 635; most sensitive: Zheda 622) to investigate how SeNPs affect stomatal function under Cu stress exposure (Figure 2C,D). Results demonstrated that Cu (200 µM) toxicity caused substantial morphological deterioration of the stomatal structures in *B. napus* plants. In contrast, the application of SeNPs significantly reduced the structural abnormalities induced by Cu in the stomatal complex. Nevertheless, SeNPs (25 µM) treatments displayed considerably wider stomatal openings relative to those experiencing Cu stress without SeNPs intervention. Importantly, the Zheda 635 (most tolerant) exhibited more pronounced protective benefits than the Zheda 622 (most sensitive) as shown in Figure 2C,D. These findings establish that Zheda 635 possesses substantial capacity to counteract Cu-mediated disruption of stomatal regulatory mechanisms in *B. napus*.

### 2.4. Impact of Exogenous SeNPs and Cu on Oxidative Damage

**Figure 4:**
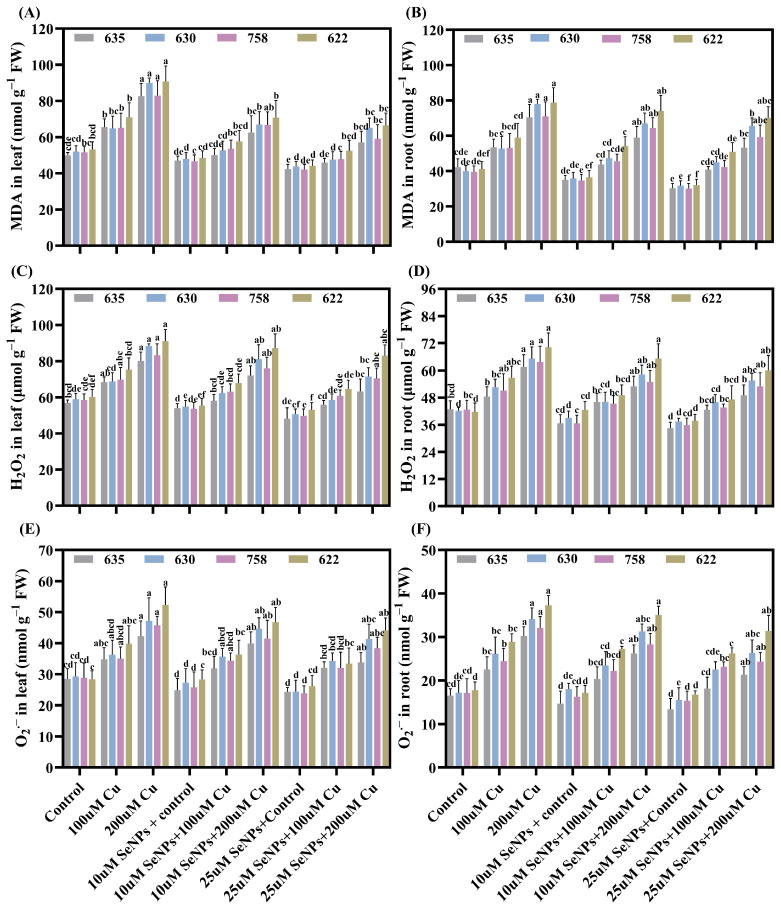
Synergistic effects of copper (0, 100, and 200 μM) and exogenous SeNPs (10 and 25 μM) and their interactions on reactive oxygen species (ROS) in leaf and roots of *B. napus* cultivars (Zheda 635, Zheda 630, ZS 758, and Zheda 622): (**A**) MDA in leaf, (**B**) MDA in roots, (**C**) H_2_O_2_ in leaf, (**D**) H_2_O_2_ in roots, (**E**) O_2_^•–^ in leaf, and (**F**) O_2_^•–^ in roots. Bars are presented as mean ± SD (*n* = 3). Bars sharing the same lowercase letter are not significantly different, whereas bars with different letters differ significantly according to Tukey’s HSD test at *p* ≤ 0.05, with comparisons made within each cultivar.

Copper exposure concomitantly elevated the generation of ROS, including hydrogen peroxide (H_2_O_2_) and superoxide radical (O_2_^•–^), and increased malondialdehyde (MDA) content in leaf and roots of *B. napus* (Figure 4A–F). Compared to control specimens, Cu (200µM) stressed plants exhibited substantial increases in MDA, indicative of extensive oxidative damage and membrane lipid peroxidation. Copper at 200 μM significantly elevated MDA, H_2_O_2_, and O_2_^•−^ in both leaf and root tissues across all cultivars, with root tissues showing more pronounced accumulation than leaves. Zheda 622 exhibited the highest oxidative stress markers while Zheda 635 showed the least accumulation in the corresponding cultivars under Cu stress conditions (Figure 4E,F). Taken together, these findings indicate that Zheda 635 is the most tolerant cultivar with the least ROS accumulation in all parameters, while 622 is the most sensitive with the highest accumulations of oxidative stress in both tissues.

In contrast, SeNP treatment significantly reduced MDA, H_2_O_2_, and O_2_^•−^ levels in both leaf and root tissues, thereby preserving membrane integrity. Conversely, the combined application of SeNPs (25 µM) under Cu (200 µM) exposure markedly attenuated ROS generation relative to Cu treatment alone (Figure 4A–F). Compared with the Cu-stressed plants, MDA content in foliar tissue exhibited significant reductions (by 30.80%, 27.62%, 28.66%, and 26.80%) as well as decreases in root tissue (of 24.42%, 15.85%, 16.35%, and 10.96%) in Zheda 635, Zheda 630, ZS 758, and Zheda 622, respectively. H_2_O_2_ content in leaves decreased (by 21.03%, 19.09%, 15.33%, and 8.78%), while that in roots also declined (20.38%, 14.95%, 16.81%, and 14.35%) in the corresponding cultivars. In addition, the concentration of O_2_^•−^ in leaves was lower (by 19.89%, 12.30%, 16.23%, and 15.64%), and decreases were also exhibited in roots (of 29.45%, 22.75%, 24.17%, and 15.91%) in the same cultivars. Taken together, these results suggested that all cultivars were severely subjected to Cu-induced oxidative stress, which was greatly ameliorated by SeNPs.

### 2.5. Impact of Exogenous SeNPs and Cu on Nutrient Content

Exposure to Cu led to substantial decreases in nutrient accumulation (Fe, P, and K), with leaf and root tissue concentrations declining notably across multiple elements (Figure S4). Copper exposure significantly reduced Fe, P, and K contents in both leaf and root tissues of the most tolerant (Zheda 635) most sensitive (Zheda 622) cultivars (Figure S4A–F). When SeNPs (25 μM) administered individually under Cu stress proved substantially effective at restoring nutrient homeostasis. This dual treatment enhanced nutrient accumulation in leaf increased by 34.17% and 21.70% in Fe, 35.32% and 26.54% in P, and 17.55% and 27.56% in K, and in roots by 18.15% and 15.71% in Fe, 15.79% and 14.49% in P, and 25.83% and 23.59% in K, in Zheda 635 and Zheda 622 respectively, compared to plants receiving only Cu treatment (Figure S4A–F). The SeNPs treatment proved effective at maintaining mineral balance in *B. napus* by suppressing Cu accumulation and preserving nutrient uptake in both leaves and roots.

### 2.6. Impact of Exogenous SeNPs and Cu on Volatile Organic Compound (VOC) in B. napus Cultivars

**Figure 5:**
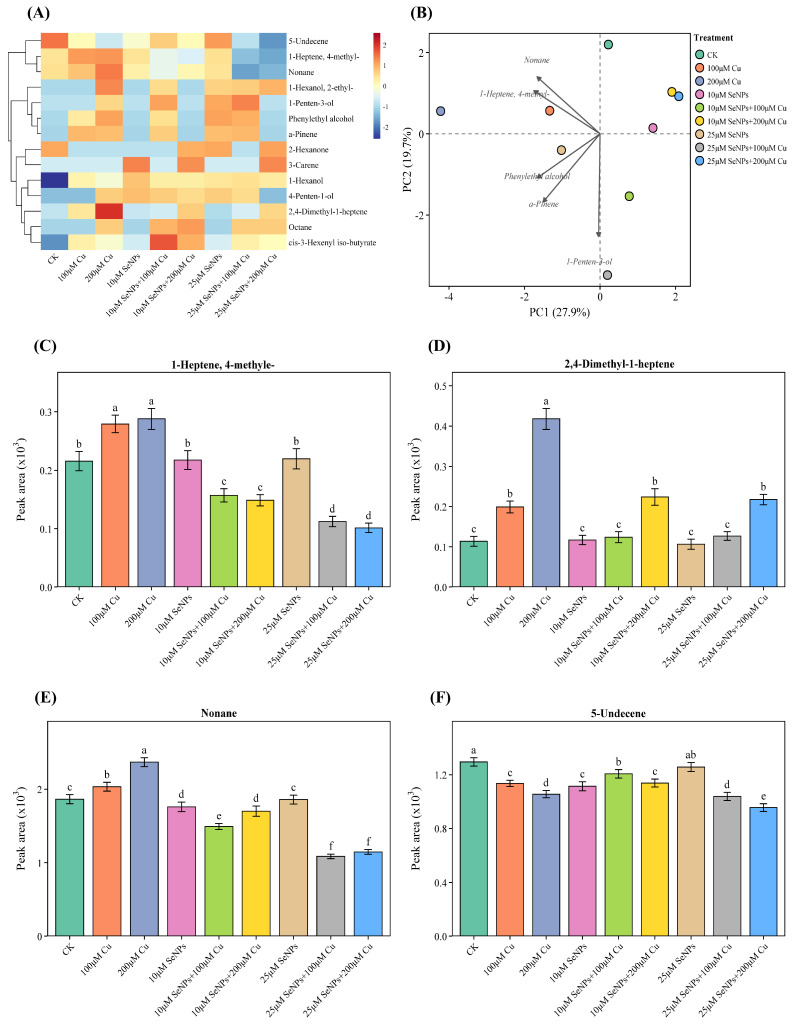
Synergistic effects of copper (0, 100, and 200 μM) and exogenous SeNPs (10 and 25 μM) and their interactions markedly modify the metabolite composition of *B. napus* cultivar Zheda 635 (most tolerant). (**A**) Heatmap visualization shows clear variation in metabolite abundance among the treatment groups. Metabolite levels are expressed as log_2_ intensities, where red represents higher abundance and dark blue indicates lower abundance. (**B**) Principal component analysis (PCA) of metabolomic data reveals clustering of samples according to treatment, reflecting treatment-specific metabolic profiles. Changes in the relative concentrations of selected key volatile organic compounds (VOCs) under different treatments: (**C**) 1-Heptene, 4-methyl-, (**D**) 2,4-Dimethyl-1-heptene, (**E**) Nonane, and (**F**) 5-Undecene. Columns labeled with different letters denote significant differences among treatments as determined by ANOVA followed by Tukey’s HSD test (*p* < 0.05).

**Figure 6:**
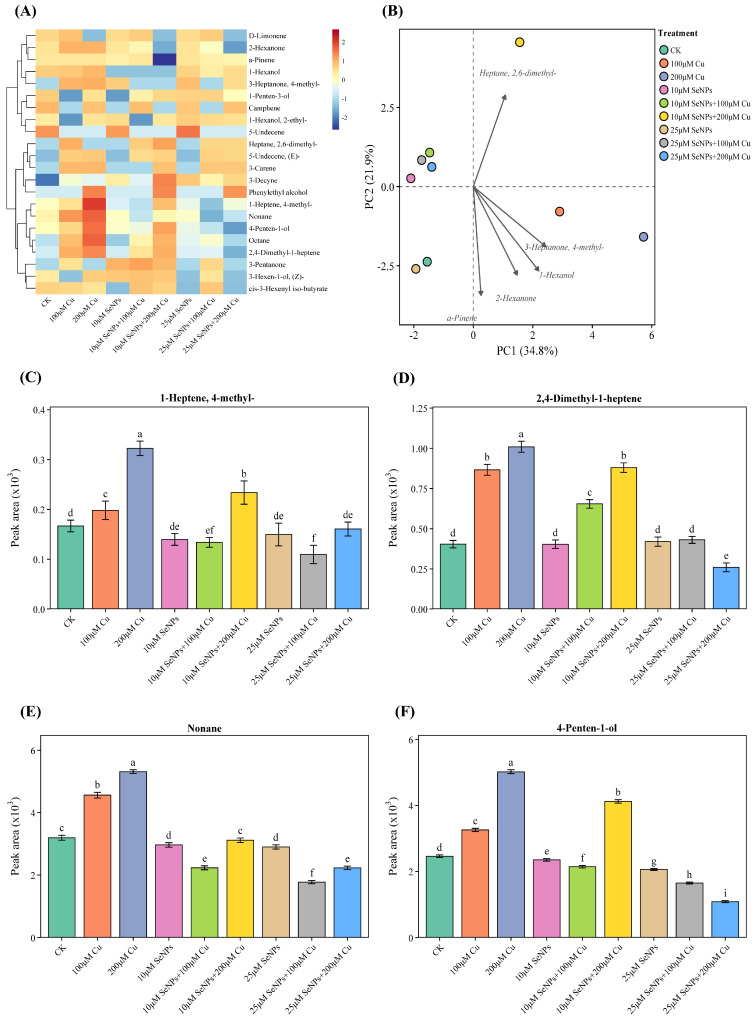
Synergistic effects of copper (0, 100, and 200 μM) and exogenous SeNPs (10 and 25 μM) and their interactions markedly modify the metabolite composition of *B. napus* cultivar Zheda 622 (most sensitive). (**A**) Heatmap visualization shows clear variation in metabolite abundance among the treatment groups. Metabolite levels are expressed as log_2_ intensities, where red represents higher abundance and dark blue indicates lower abundance. (**B**) Principal component analysis (PCA) of metabolomic data reveals clustering of samples according to treatment, reflecting treatment-specific metabolic profiles. Changes in the relative concentrations of selected key volatile organic compounds (VOCs) under different treatments: (**C**) 1-Heptene, 4-methyl-, (**D**) 2,4-Dimethyl-1-heptene, (**E**) Nonane, and (**F**) 4-Penten-1-ol. Columns labeled with different letters denote significant differences among treatments as determined by ANOVA followed by Tukey’s HSD test (*p* < 0.05).

This study investigated the impact of SeNPs, Cu, and their combination on the secondary metabolite profile in *B. napus* cultivars, the most tolerant Zheda 635 (Figure 5), and the most sensitive Zheda 622 (Figure 6). The heatmap analysis effectively demonstrated distinct treatment-dependent patterns of individual metabolite accumulation between the two cultivars. Overall, Zheda 635 had a relatively lower expression level of most VOCs across all treatments (Figure 5A), and Zheda 622 had a higher and more diverse pattern of metabolites accumulated, suggesting a significant cultivar difference in secondary metabolism and stress response (Figure 6A). Principal component analysis (PCA) also revealed the distinct metabolic behaviors between the two cultivars. In Zheda 635, PC1 (27.9%) and PC2 (19.7%) together represented a relatively moderate proportion of the total variance, and partial overlap was observed between the treatment groups (Figure 5B). This implies a relatively stable metabolic profile and limited reprogramming among the treatments. In Zheda 622, PC1 (34.8%) and PC2 (21.9%) represented a significantly higher proportion of the total variance, and the treatments were clearly separated along these axes (Figure 6B). Analysis of the accumulation of individual VOC also revealed treatment-specific patterns in both cultivars. In Zheda 635, 1-heptene 4-methyl- accumulated at significantly higher levels in response to Cu-related treatments, with a significant difference between Cu alone and combined treatments (Figure 5C). Also, 2,4-Dimethyl-1-heptene showed its highest accumulation in Cu (200 μM) treatment, related to other treatments (Figure 5D). This demonstrated a significant and specific response to Cu stress. Nonane showed the highest (almost double that of other treatments) expression under Cu (200 μM) of Zheda 635 (Figure 5E). Expression under control and combined treatments was relatively lower in comparison to Cu stress alone.

In Zheda 622, metabolite accumulation patterns were more dynamic and pronounced. Further, 1-heptene 4-methyl- was significantly upregulated under Cu-related treatment, with the highest expression observed under Cu (200 μM) (Figure 6C). Expression levels under these treatments were found to be significantly higher than those of the control and combined treatments. Furthermore, 2,4-Dimethyl-1-heptene showed the maximum accumulation under Cu treatment, followed by the control, while the combined treatments resulted in relatively lower levels (Figure 6D). Nonane showed maximum expression under Cu treatment, while elevated levels were also found as compared to the control and combined treatments (Figure 6E). Furthermore, 4-penten-1-ol was found to accumulate the most under Cu treatment, intermediate levels under combined SeNPs + Cu exposure (Figure 6F). Cultivar comparison shows that the absolute level of each metabolite was higher in Zheda 622, and the responses to treatments were more variable than in Zheda 635. Differences between cultivars were less pronounced in Zheda 635, which exhibited more moderate and stable responses to Cu and SeNPs treatments. In both cultivars, most metabolites were at intermediate levels in the combined SeNPs + Cu treatments compared to single stress treatments, suggesting an interaction between SeNPs and Cu in the regulation of secondary metabolites.

### 2.7. Impact of Exogenous SeNPs and Cu on Antioxidant Activity

**Figure 7:**
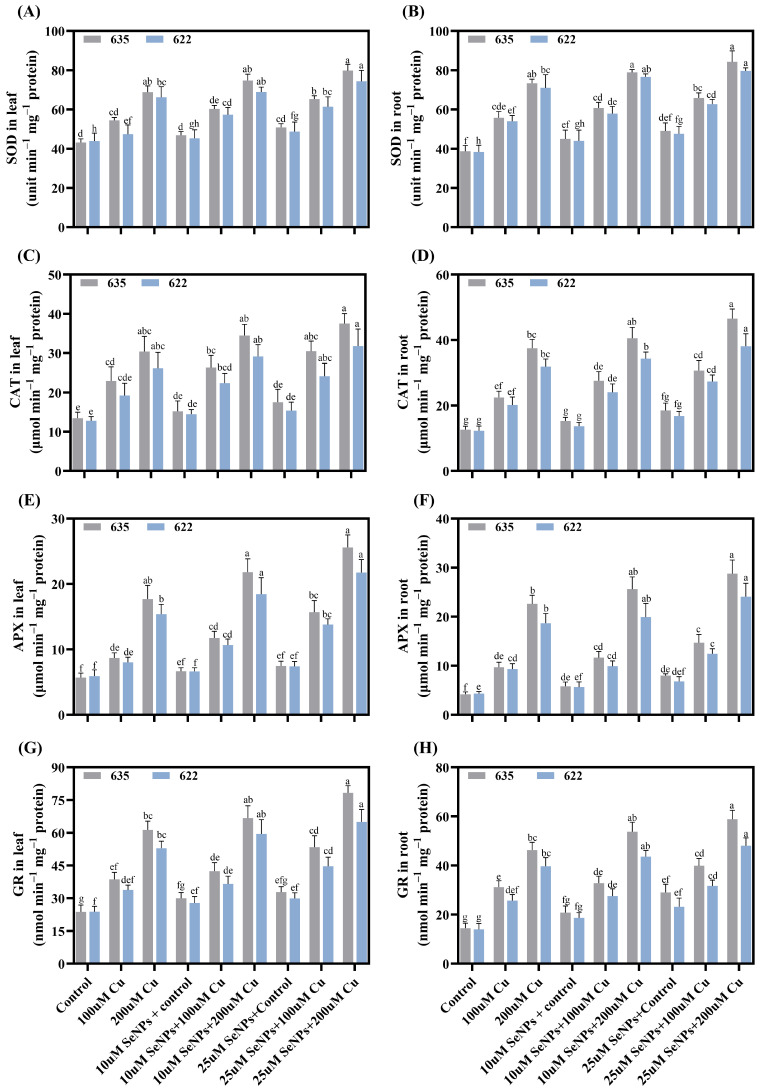
Synergistic effects of copper (0, 100, and 200 μM) and exogenous SeNPs (10 and 25 μM) and their interactions on antioxidants enzymes activity in leaf and root of *B. napus* cultivars in Zheda 635 (most tolerant) and Zheda 622 (most sensitive): (**A**) SOD in leaf, (**B**) SOD in root, (**C**) CAT in leaf, (**D**) CAT in root, (**E**) APX in leaf, (**F**) APX in root, (**G**) GR in leaf, and (**H**) GR in root. Bars are presented as mean ± SD (*n* = 3). Bars sharing the same lowercase letter are not significantly different, whereas bars with different letters differ significantly according to Tukey’s HSD test at *p* ≤ 0.05, with comparisons made within each cultivar.

Exposure to Cu induced substantial modifications in antioxidant enzyme activities across leaf and root tissues of the most tolerant (Zheda 635) and most sensitive (Zheda 622) *B. napus* cultivars (Figure 7A–H). Copper at 200 μM markedly elevated superoxide dismutase (SOD), catalase (CAT), ascorbate peroxidase (APX), and glutathione reductase (GR) activities in both leaf and root tissues across both cultivars, with APX showing the highest induction and root tissues exhibiting greater enzyme activities than leaves. Supplementation with 25 µM SeNPs further augmented the activities of SOD by 15.99% and 12.39%, CAT by 23.47% and 21.43%, APX by and 44.51% and 41.34%, and GR by 27.80% and 22.92% in leaves, in Zheda 635 and Zheda 622 respectively. Similarly, root tissues demonstrated enhancement with SOD elevated by 14.84% and 12.31%, CAT by 24.24% and 19.48%, APX by and 27.17% and 28.74%, and GR by 27.12% and 20.97%, respectively, beyond the response observed under 200 µM Cu stress alone (Figure 7A–H). Notably, SeNPs application effectively modulated antioxidant enzyme activities and also amplifying the stress-induced responses while restoring suppressed enzymatic functions. This demonstrates comprehensive regulation of the antioxidant enzymatic network under metal stress conditions.

### 2.8. Impact of Exogenous SeNPs and Cu on Antioxidant Gene Expression

**Figure 8:**
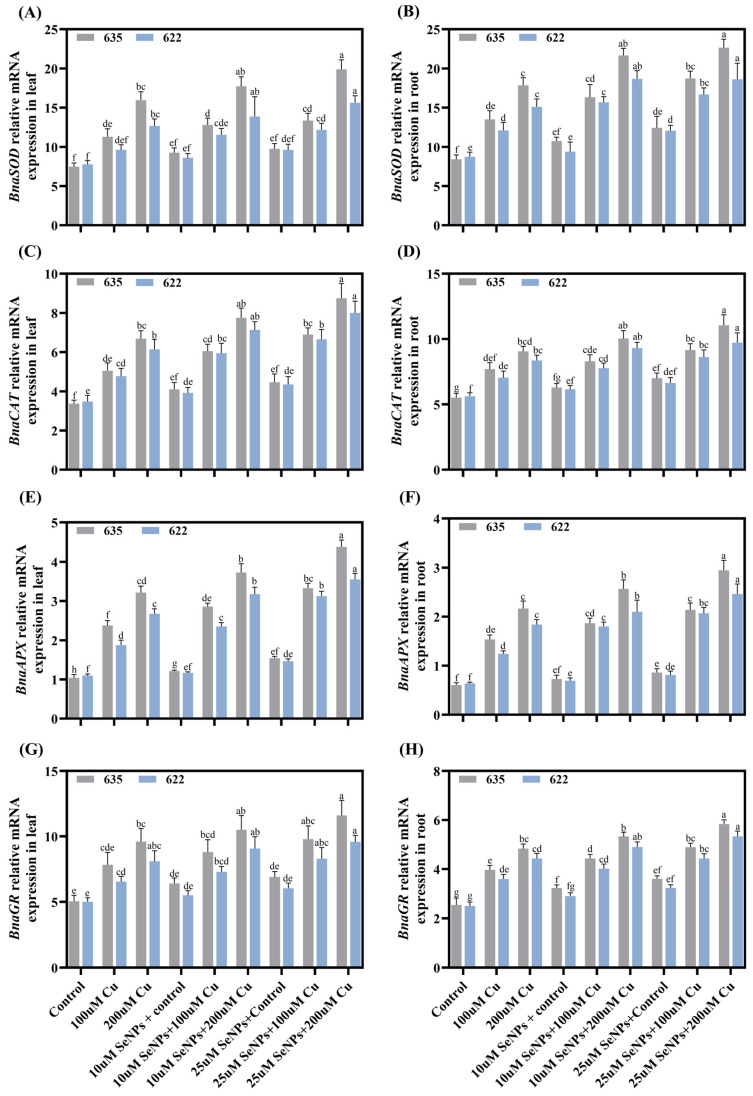
Synergistic effects of copper (0, 100, and 200 μM) and exogenous SeNPs (10 and 25 μM) and their interactions on gene expression of antioxidant enzymes in leaf and root of *B. napus* cultivars in Zheda 635 (most tolerant) and Zheda 622 (most sensitive): (**A**) *BnaSOD* in leaf, (**B**) *BnaSOD* in root, (**C**) *BnaCAT* in leaf, (**D**) *BnaCAT* in root, (**E**) *BnaAPX* in leaf, (**F**) *BnaAPX* in root, (**G**) *BnaGR* in leaf, and (**H**) *BnaGR* in root. Bars are presented as mean ± SD (*n* = 3). Bars sharing the same lowercase letter are not significantly different, whereas bars with different letters differ significantly according to Tukey’s HSD test at *p* ≤ 0.05, with comparisons made within each cultivar.

Transcript abundance of antioxidant-encoding genes, specifically *BnaSOD*, *BnaCAT*, *BnaAPX,* and *BnaGR,* was substantially upregulated within both leaf and root tissues of *B. napus* (most tolerant, Zheda 635; most sensitive, Zheda 622) cultivars exposed to 200 μM Cu, as revealed by qRT-PCR analysis (Figure 8A–H). The magnitude of transcript elevation observed under 100 μM Cu exposure was markedly lower relative to that documented at the 200 μM Cu concentration. Among all antioxidant genes, APX exhibited the highest transcriptional upregulation under 200 μM Cu stress in both leaf and root tissues, followed by SOD, GR, and CAT. Zheda 635 consistently showed greater fold increases across all measured genes compared to Zheda 622, reflecting its superior transcriptional response to Cu-induced oxidative stress. Application of SeNPs further intensified these transcriptional responses of antioxidant gene expression. The synchronized elevation of gene transcription represents a comprehensive defense strategy against Cu mediated oxidative damage. This coordinated transcriptional response across both leaf and root tissues suggests the involvement of upstream stress-responsive regulatory networks governing antioxidant gene expression in a cultivar-dependent manner.

### 2.9. Impact of Exogenous SeNPs and Cu on Tissue Cu and Se Content

**Figure 9:**
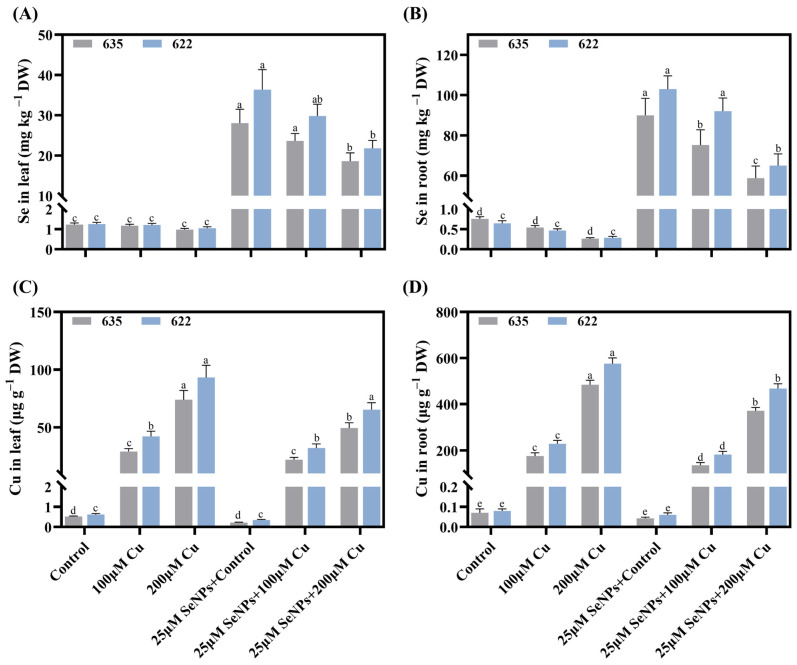
Synergistic effects of copper (0, 100, and 200 μM) and exogenous SeNPs (25 μM) and their interactions on accumulation of selenium (Se) and copper (Cu) content in leaf and root of *B. napus* cultivars in Zheda 635 (most tolerant) and Zheda 622 (most sensitive): (**A**) Se in leaf, (**B**) Se in root, (**C**) Cu in leaf, and (**D**) Cu in root. Bars are presented as mean ± SD (*n* = 3). Bars sharing the same lowercase letter are not significantly different, whereas bars with different letters differ significantly according to Tukey’s HSD test at *p* ≤ 0.05, with comparisons made within each cultivar.

Exogenous application of SeNPs (25 μM) significantly increased the levels of endogenous Se in leaf and root tissues, suggesting that these compounds might be involved in the stress defense system (Figure 9A,B). The application of Cu (200 μM) reduced the levels of endogenous Se content in leaves (by 20.05% and 16.26%) and in roots (by 65.35% and 55.15%) in Zheda 635 and Zheda 622, respectively, compared to the control. In contrast, treatment with SeNPs (25 μM) under Cu stress conditions significantly increased the levels of endogenous Se in leaves (by 33.57% and 40.05%) and roots (by 34.67% and 36.85%), respectively, compared to Cu stress (Figure 9A,B). The Cu treatment alone significantly increased Cu contents in leaves (by 73.82 and 93.05 μg g^−1^) and in roots (by 484.28 and 575.68 μg g^−1^) compared to the control groups (Figure 9C,D). The application with SeNPs significantly decreased Cu accumulation in leaves (by 33.04% and 29.96) and in roots (by 23.39% and 18.72%), respectively, relative to Cu application.

### 2.10. Principal Component Analysis (PCA) of Genotypic Responses

**Figure 10:**
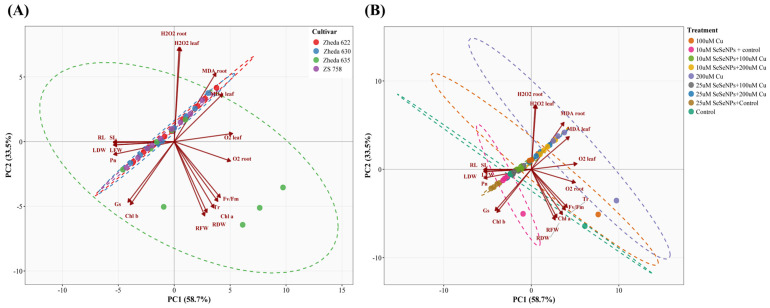
Physiobiochemical principal component analysis (PCA) biplot of *B. napus* cultivars under copper (0, 100, and 200 μM) and exogenous SeNPs (10 and 25 μM) and their interactions. (**A**) PCA biplot illustrating the distribution and clustering of *B. napus* cultivars (Zheda 635, Zheda 630, ZS 758, and Zheda 622) based on combined physiobiochemical attributes. (**B**) PCA biplot showing the separation of samples according to different treatments, including Cu stress (0, 100, and 200 µM), exogenous SeNPs (10 and 25 µM), and their combined applications.

PCA was performed on 18 physiological traits across 36 cultivars × treatment combinations to identify the major axes of phenotypic variation under Cu stress and SeNPs supplementation (Figure 10A,B). The first two principal components collectively accounted for 92.2% of the total variance (PC1: 58.7%; PC2: 33.5%), indicating that the multivariate dataset could be adequately represented in two dimensions (Table S3). Only PC1 and PC2 had eigenvalues substantially exceeding unity (10.87 and 6.20, respectively), satisfying the Kaiser criterion. PC1 was primarily driven by growth and biomass traits, with the highest positive loadings on LDW (0.301, contributing 9.05%), RL (0.301, 9.04%), SL (0.300, 9.02%), LFW (0.300, 9.00%), and Pn (0.300, 8.97%) (Table S4). Oxidative damage markers loaded negatively on PC1, with O_2_^•–^ in leaf (−0.289, 8.35%) and root (−0.279, 7.78%) showing the strongest negative contributions. Thus, PC1 represents a growth-versus-oxidative-stress gradient, where positive scores indicate vigorous growth and negative scores indicate stress-induced damage. PC2 was dominated by ROS accumulation and root morphological traits. H_2_O_2_ in root (+0.403, 16.27%) and leaf (+0.402, 16.15%) together contributed 32.4% of the PC2 variance, followed by RFW (−0.318, 10.10%), RDW (−0.303, 9.20%), and MDA in root (+0.294, 8.63%). The opposing signs of H_2_O_2_ (positive) and root biomass traits (negative) on PC2 indicate that this axis captures the antagonistic relationship between ROS accumulation and root development, independent of overall growth performance.

Notably, several traits contributed minimally to PC1 while being major drivers of PC2, and vice versa. H_2_O_2_ in leaf and root had near-zero PC1 contributions (0.09% and 0.06%, respectively) but dominated PC2 (16.15% and 16.27%), whereas SL and RL strongly contributed to PC1 (9.02% and 9.04%) but had negligible PC2 contributions (<0.01%). This orthogonal partitioning confirms that growth impairment and ROS accumulation represent biologically distinct dimensions of Cu stress response in *B. napus*. The cultivar-based PCA revealed distinct clustering patterns, with Zheda 635 exhibiting pronounced separation from other genotypes, suggesting differential physiological and oxidative stress responses (Figure 10A). The treatment-based PCA demonstrated clear segregation between Cu-alone treatments, strongly associated with oxidative damage indicators, and SeNPs-amended treatments distributed along negative PC1, aligning with growth attributes and photosynthetic performance (Figure 10B).

## 3. Discussion

The present study demonstrates that SeNPs effectively mitigate Cu-induced phytotoxicity in multiple *B. napus* cultivars through coordinated improvements in growth, photosynthetic efficiency, secondary metabolite profiles, and antioxidant defense systems. The spherical SeNPs with high purity provide an optimal platform for plant stress mitigation [25,26], as their small particle size and uniform distribution are critical factors influencing NPs bioavailability and plant uptake efficiency [27]. Previous studies have demonstrated that NPs with a smaller particle size exhibit enhanced cellular penetration and bioactivity compared to larger particles [28], which likely contributes to the protective effects observed in the present study. Copper exposure caused severe growth inhibition across all *B. napus* cultivars, with reductions in leaf and root biomass varying depending on the cultivar and parameter measured. The differential sensitivity among cultivars, with Zheda 622 showing the most severe growth suppression and Zheda 635 demonstrating relatively greater tolerance, reflects cultivar variation in Cu tolerance mechanisms. These growth reductions are consistent with well-documented Cu phytotoxicity mechanisms, including disruption of nutrient uptake and interference with metabolic processes [29,30,31]. Specifically, Cu competes with essential nutrients such as Fe, Zn, and Mn for transporter proteins, thereby disrupting mineral homeostasis and impairing metabolic functions critical for cell division and biomass accumulation [7,8,10]. The application of SeNPs (25 μM) substantially alleviated these growth inhibitions, suggesting that the NPs interact with multiple physiological processes to restore normal plant development [3,32,33]. Root Cu concentrations decreased most substantially in Zheda 635, the more tolerant cultivars, suggesting that these genotypes possess an enhanced capacity to restrict Cu uptake or promote Cu efflux when Se availability is improved. The observed reduction in Cu concentrations in both shoot and root tissues following SeNPs co-application suggests that SeNPs interfere with Cu availability and uptake, although the precise mechanism remains to be fully established. This reduction may potentially involve Se-Cu antagonism at the root uptake level, where Se and Cu compete for similar transporter proteins, thereby limiting Cu entry into plant tissues. Similarly, [34] reported that the application of MnNPs alleviated As stress by reducing As availability and enhancing Mn availability in five *B. napus* genotypes.

The simultaneous increase in tissue Se concentrations under SeNPs + Cu treatment compared to Cu-alone treatment demonstrates that exogenously applied SeNPs successfully restore Se nutritional status. These physiological processes serve to restrict excessive translocation of the Cu to leaf tissues while maintaining adequate concentrations in root tissues for essential metabolic functions. SeNPs likely influence nutrient uptake through multiple interconnected mechanisms, including competition with Cu for shared transporter proteins at the root surface, thereby reducing excessive Cu influx while maintaining the availability of essential nutrients. Furthermore, SeNPs may stabilize root cell membrane integrity by reducing lipid peroxidation, thereby preserving the structural and functional properties of nutrient transporters. The restoration of cellular redox homeostasis by SeNPs may also indirectly support active nutrient uptake processes that are otherwise impaired under Cu-induced oxidative stress, as evidenced by the significant recovery of nutrient contents observed in the present study. The severe reductions in Chl content, Pn, and PSII (Fv/Fm) under Cu stress had reflected the metal’s multifaceted disruption of photosynthetic machinery [35,36]. The concurrent decreases in gs and Tr indicate that Cu toxicity extends beyond biochemical photosynthetic limitations to include stomatal dysfunction. This impairment likely results from disrupted guard cell signaling within the plants. The application of SeNPs remarkably restored these photosynthetic parameters. Consistent with these findings, [37] demonstrated that SeNPs restored CO_2_ fixation and photosynthetic efficiency in metal-stressed algae, suggesting a conserved mechanism of SeNP-mediated photosynthetic protection across different plant systems. This suggests that the restoration of chlorophyll content occurs because SeNPs either protect chlorophyll biosynthesis enzymes from Cu-induced inhibition or facilitate the replacement of damaged chlorophyll molecules. The recovery of Fv/Fm ratios indicates protection of PSII reaction centers, which are particularly vulnerable to metal-induced oxidative damage [38,39]. Microscopic examination of stomatal structure provided visual confirmation of the protective effects of SeNPs, revealing restoration of guard cell morphology and stomatal aperture. However, this restoration exhibits cultivar-dependent responses. These differential effects suggest that SeNPs influence ion homeostasis and osmotic regulation in guard cells, processes that are disrupted by metal accumulation [21,40]. This restoration of stomatal function is critical for maintaining the water balance and gas exchange necessary for sustained photosynthetic activity and overall plant metabolism.

The substantial increases in antioxidant activities under Cu stress reflect activation of the primary enzymatic antioxidant network in response to Cu-induced ROS generation [41], representing an active biochemical strategy to counteract oxidative damage. The higher enzyme activities in roots compared to leaves align with roots being the primary site of Cu uptake and accumulation, thus experiencing more severe oxidative stress [36]. The coordinated upregulation of these enzymes represents an integrated defense strategy. In this system, SOD converts superoxide radicals to H_2_O_2_, subsequently detoxified by CAT and APX, and GR regenerates the reduced glutathione necessary for the ascorbate-glutathione cycle [42]. This creates a comprehensive detoxification system that processes ROS through multiple complementary pathways. The further enhancement of enzyme activities following SeNPs + Cu co-treatment demonstrates that SeNPs actively potentiate antioxidant defenses rather than merely preventing ROS generation. Consistent with our findings, [3] reported that MT-SeNPs markedly enhanced antioxidant enzyme activities in *B. napus* under As stress. These findings indicate that SeNPs play an active regulatory role in strengthening enzymatic antioxidant defense systems. Although the precise regulatory mechanisms governing this transcriptional upregulation were not directly investigated in the present study, the coordinated induction of *BnaSOD*, *BnaCAT*, *BnaAPX,* and *BnaGR* likely reflects activation of ROS-responsive signaling networks. SeNPs may further modulate these upstream regulatory networks by optimizing cellular redox homeostasis, thereby sustaining transcriptional activation of antioxidant defense genes. Future studies incorporating transcriptomic and signaling pathways analysis would be warranted to elucidate the precise molecular mechanisms underlying SeNPs-mediated antioxidant gene regulation.

The cultivar-specific responses to treatment, with Zheda 635 showing the most robust enzymatic enhancement and Zheda 622 the weakest, parallel the observed patterns in growth and physiological tolerance. This correlation indicates that a plant’s inherent antioxidant capacity is a key determinant of its overall metal tolerance [43]. These enhanced antioxidant defenses lead to reduced oxidative damage [44]. This reduction is evidenced by the elevated levels of ROS under Cu stress, which provide direct evidence of oxidative damage to cellular membranes. SeNP treatment significantly reduced these oxidative stress markers. The particularly strong responses in cultivars Zheda 630 and Zheda 622 for oxidative damage mitigation suggest that SeNPs may partially compensate for inherent weaknesses in these sensitive cultivars’ stress defense systems. For example, cultivar-dependent stress mitigation by NPs has been documented by [45], who demonstrated that CuNPs significantly reduced oxidative damage and enhanced antioxidant capacity under Cd stress in *B. napus* cultivars. These findings indicate that NPs application can partially compensate for inherent deficiencies in plant stress defense systems.

The contrasting secondary metabolite profiles of the tolerant cultivar Zheda 635 and the sensitive cultivar Zheda 622 demonstrate strong genotype-dependent metabolic adaptations in response to Cu stress and SeNPs application. The lower metabolite abundance and reduced variability observed in Zheda 635 suggest reliance on efficient primary defense mechanisms that minimize the need for extensive activation of secondary metabolism [34,38]. In contrast, Zheda 622 exhibited markedly higher accumulation of VOCs, indicating a stronger induction of stress-responsive metabolic pathways. Many of the compounds elevated in Zheda 622, are associated with lipid oxidation processes related to the lipoxygenase pathway [46,47]. Biochemically, the elevated VOCs observed in Zheda 622 are products of lipoxygenase-mediated oxidation of membrane polyunsaturated fatty acids, directly reflecting greater membrane lipid peroxidation and compromised cellular integrity under Cu stress. In contrast, the lower VOC accumulation in Zheda 635 indicates better maintenance of membrane stability and more efficient ROS detoxification, consistent with its superior antioxidant enzyme activities observed in the present study. Under stress conditions, the lipoxygenase pathway is activated, causing the breakdown of membrane lipids and resulting in the formation of VOCs [48]. Their enhanced accumulation in the Zheda 622 likely reflects greater cellular damage under Cu exposure and represents a compensatory defense response involved in stress signaling and protection. The more moderate induction of these metabolites in Zheda 635 suggests better maintenance of membrane integrity and redox balance. Differences in PCA patterns further support distinct metabolic strategies between the cultivars. The strong treatment separation and high variance explained in Zheda 622 indicate pronounced metabolic plasticity and extensive reorganization under Cu stress, particularly under combined SeNPs + Cu exposure. In contrast, the overlapping clusters and lower variance in Zheda 635 reflect metabolic stability and targeted stress responses, traits commonly associated with stress tolerance [34].

The observation that combined SeNPs + Cu treatments resulted in intermediate metabolite levels in both cultivars indicates that SeNPs modulate rather than intensify Cu-induced metabolic responses. This effect may arise from reduced Cu uptake or enhanced antioxidant capacity, thereby limiting oxidative damage and subsequent VOCs production. A consistent pattern of cultivar-specific responses is evident across all measured parameters, with Zheda 635 generally showing the greatest tolerance and Zheda 622 the greatest sensitivity. This indicates that SeNPs supplementation could serve as both a remediation strategy for sensitive cultivars and an enhancement strategy for tolerant ones. The cultivar-specific responses observed in this study underscore the strategic importance of integrating SeNPs application with appropriate genotype selection for optimizing crop performance under varying Cu contamination levels. The PCA loading structure provided further mechanistic insight into the multivariate nature of Cu stress responses and SeNPs-mediated alleviation. The orthogonal separation of growth traits on PC1 from H_2_O_2_ accumulation patterns on PC2 indicates that Cu-induced growth suppression and ROS accumulation represent two biologically independent dimensions of stress response in *B. napus*, consistent with the dual protective role of SeNPs in simultaneously restoring photosynthetic capacity and biomass accumulation while modulating ROS homeostasis [25,26]. The near-equal contributions of growth traits to PC1 suggest a tightly coordinated growth response, wherein SeNPs supplementation simultaneously recovered shoot growth, root elongation, and photosynthetic efficiency. Furthermore, the cultivar-dependent positioning along PC2 indicates that tolerant cultivars such as Zheda 635 maintained more effective H_2_O_2_ scavenging relative to root biomass, consistent with their superior antioxidant enzyme activities observed in the present study [34].

The findings at the vegetative stage are particularly important because early physiological and biochemical disruptions under metal stress are well-known to translate into later reductions in crop productivity. The consistent pattern of cultivar-specific responses observed across all measured parameters in the present study clearly highlights that Zheda 635 and Zheda 622 employ fundamentally different strategies to cope with Cu stress. Zheda 635 relies on efficient primary antioxidant defense mechanisms with metabolic stability, whereas Zheda 622 depends more heavily on secondary metabolic reprogramming and extensive VOC accumulation to counteract Cu-induced damage. These contrasting strategies have important practical implications, as they suggest that genotype selection should be carefully considered when applying SeNPs as a remediation strategy in Cu-contaminated agricultural soils.

Although the present study focused on the vegetative stage, the physiological and biochemical disorders induced by Cu stress at this developmental stage are well-recognized as primary drivers of reduced plant production. The reduction in plant growth and production under metal stress is the final consequence of changes that initially occur at the biochemical, physiological, and mineral levels of plants [49]. Notably, reduced photosynthetic activity and chlorophyll concentration were observed under Cu stress. These physiological impairments at the vegetative stage accompanied reduced plant height and seed production, confirming that they are strong predictors of yield outcomes. The amelioration of Cu-induced disorders through SeNPs application, therefore, provides a scientifically grounded basis for safeguarding crop productivity. This is consistent with evidence that Se supplementation restores photosynthesis, antioxidant defense, and physiological attributes under metal stress, while also improving plant yield and quality [50,51,52].

Although SeNPs at 25 μM demonstrated protective effects in the present study, the potential phytotoxicity of SeNPs at higher concentrations and their long-term accumulation in plant tissues were not evaluated. Furthermore, the behavior of SeNPs in complex soil environments may differ significantly from hydroponic conditions due to interactions with soil organic matter, pH, and microbial communities, which could potentially alter soil nutrient cycling processes under repeated application scenarios. Future studies should therefore investigate the long-term fate of SeNPs in soil systems, their potential accumulation in edible plant tissues, and possible entry into the food chain under prolonged field exposure conditions to ensure their safe and sustainable agricultural application.

## 4. Materials and Methods

### 4.1. Characterization of Selenium Nanoparticle (SeNPs)

High-purity SeNPs (99%) were procured from Sigma-Aldrich Co. (St. Louis, MO, USA) and stored in sealed conditions at −80 °C. Multiple technique characterizations were employed to assess SeNP properties. Morphological examination and size determination were conducted using SEM (ZEISS GEMINI 300, Oberkochen, Germany) and TEM (Hitachi HT-7820, Tokyo, Japan) as described by [53]. Elemental analysis was performed using EDS attached to the SEM instrument. Moreover, FTIR (NICOLET iS50, Thermo Fisher Scientific, Waltham, MA, USA) spectroscopy was employed to characterize the surface functional groups of SeNPs according to [54], while diffraction patterns were analyzed by XRD (D8 Advance, Bruker, Germany) as per [55].

### 4.2. Plant Materials, Experimental Design, and Growth Conditions

Healthy seeds of *B. napus* (rapeseed cv. Zheda 635, ZS 758, Zheda 622, and Zheda 630) were procured from the College of Agriculture and Biotechnology, Zhejiang University, China. *B. napus* seeds underwent surface sterilization in 1% (*v*/*v*) sodium hypochlorite (NaClO) solution for 10 min, followed by thorough rinsing with sterile distilled water to eliminate residual sterilant (chloride or surface impurities). Sterilized seeds were aseptically arranged on moistened filter paper within sterile petri dishes and subjected for three days (72 h) incubation period at 25 °C under dark conditions to facilitate uniform germination. Uniformly germinated seedlings were initially transferred to 25% strength Hoagland nutrient solution (HNS) and cultivated for seven days to allow preliminary acclimatization. Subsequently, the seedlings were transitioned to 50% HNS for an additional 8 days to ensure gradual and progressive adaptation to full hydroponic growth conditions. The HNS was prepared (in μM) containing the following micro/macro nutrient composition: 4000 Ca(NO_3_)_2_.4H_2_O; 4000 (NH_4_)_2_SO_4_; 4000 K_2_SO_4_; 4000 KNO_3_; 1300 KH_2_PO_4_; 1000 MgSO_4_.7H_2_O; 50 Fe-EDTA; 10 H_3_BO_3_; 5 MnSO_4_.H_2_O; 5 ZnSO_4_.7H_2_O; 1 CuSO_4_.5H_2_O; and 0.5 Na_2_MoO_4_.2H_2_O [56]. Following this 15-day pre-culture period, seedlings were transferred to 100% HNS and maintained in a controlled growth chamber for the next 15 days under the photoperiod of 16 h light/8 h dark, temperature regime of 24/16 °C, relative humidity of 65 ± 5%, and light intensity of 400 μmol m^−2^ s^−1^.

Hydroponic culture solutions were continuously aerated via an aquarium air pump to sustain adequate dissolved oxygen levels throughout the experimental period. Furthermore, the nutrient solutions were entirely replenished on a weekly basis to ensure consistent and optimal nutrient availability for plant growth. At 30 days after germination, plants were subjected to Cu and SeNPs treatments for 14 days to simulate metal stress. Copper Chloride (CuCl_2_.2H_2_O) was obtained from Sigma-Aldrich Co. (St. Louis, MO, USA). Upon completion of the acclimatization phase, plants were subjected to nine (9) distinct treatment based on preliminary dose-response optimization studies: (T0) Control (CK); (T1) 100 μM Cu (alone); (T2) 200 μM Cu (alone); (T3) 10 μM SeNPs + control; (T4) 10 μM SeNPs + 100 μM Cu; (T5) 10 μM SeNPs + 200 μM Cu; (T6) 25 μM SeNPs + Control; (T7) 25 μM SeNPs + 100 μM Cu; and (T8) 25 μM SeNPs + 200 μM Cu (Table S1). Treatment concentrations and combinations were selected based on previous investigations for Cu [6,56] and SeNPs [57,58]. The experiment was arranged in a completely randomized design (CRD) comprising three independent biological replicates per treatment.

### 4.3. Estimation of Morphological Parameters

Following 14 days of treatment, each plant was carefully extracted from its nutrient solution and rinsed with deionized water to remove any adhering debris. Plants were separated into root and shoot fractions for fresh weight determination, subsequently oven-dried at 80 °C for dry weight assessment using an analytical balance (AUW220D, Shimadzu, Kyoto, Japan). Shoot length (SL) and root length (RL) were measured using a standard measuring scale, with careful attention to proper plant alignment to ensure accurate and precise readings.

### 4.4. Estimation of Light Harvesting Pigments, Gas Exchange, and Photosynthetic Efficiency of PSII

Fully expanded second leaves (top side) were used to estimate the pigment contents, including Chl a and Chl b [1]. Gas exchange parameters, such as Pn, gs, and Tr, were measured by using a portable infrared gas analyzer (LI-6400XT, LI-COR Biosciences, Lincoln, NE, USA), following the method described by [59]. All measurements were conducted between 09:00 and 11:00 h under daytime conditions to minimize temporal variability in physiological responses. Growth chamber parameters during measurements were set at the following conditions: photosynthetic photon flux density (PPFD) of 1000 μmol m^−2^ s^−1^ and CO_2_ concentration of 400 μmol mol^−1^. The maximum efficiency of photosystem II (Fv/Fm) was estimated with a pulse-amplitude-modulated (PAM) chlorophyll fluorometer, following 30 min dark adaptation [55].

### 4.5. Scanning Electron Microscopy (SEM) of Stomatal Opening

SEM samples of leaf tissue fragments (midrib removed) were placed in glutaraldehyde solution (2.5% in 100 mM PBS, pH 7.0) for 24 h at 4 °C. Leaf samples were then washed thrice in PBS (15 min per wash) and post-fixed with 1% osmium tetroxide for 2 h at room temperature, followed by buffer rinsing. Sequential dehydration was performed using graduated ethanol concentrations (30%, 50%, 70%, 80%, 90%, and 95%) for 15 min each, with final dehydration accomplished through 20 min immersions in 100% ethanol. Processed specimens were affixed to aluminum stubs via carbon adhesive tape, metallized with gold-palladium by ion sputtering (5 min), and analyzed using SEM. Stomatal responses were analyzed through SEM using the approach of [60,61].

### 4.6. Quantification of Oxidative Stress Markers

Quantitative assessment of lipid peroxidation as MDA, along with H_2_O_2_ and O_2_^•–^ contents, was conducted in both leaf and root tissues, employing well-established spectrophotometric methodologies [62,63,64]. Fresh tissue samples (0.5 g) were homogenized in 5 mL of ice-cold 50 mM phosphate buffer (pH = 7.0) and subjected to centrifugation at 12,000× *g* for 15 min at 4 °C. The resultant supernatant was retained for biochemical assays. Lipid peroxidation was evaluated through MDA quantification using the thiobarbituric acid reactive substances (TBARS) assay. H_2_O_2_ concentration was determined spectrophotometrically via the potassium iodide (KI)-mediated colorimetric method, with absorbance monitored at 390 nm. The rate of O_2_^•−^ generation was assessed by measuring the reduction of nitroblue tetrazolium (NBT) at 530 nm.

### 4.7. Quantification of Nutrient Contents

Following oven-drying, leaf and root samples underwent acid digestion using a mixture of HNO_3_ and HClO_4_ (5:1, *v*/*v*). Mineral nutrient contents from acid-digested samples were subjected to elemental analysis according to [65]. Copper, Se, iron (Fe), phosphorus (P), and potassium (K) concentrations were measured using inductively coupled plasma optical emission spectrometry (ICP-OES; Optima 8000DV, PerkinElmer, Waltham, MA, USA).

### 4.8. Analysis of Secondary Metabolites (Volatile Organic Compounds)

Leaf tissue from *B. napus* cultivars (Zheda 635 and Zheda 622) was harvested and immediately flash-frozen in liquid nitrogen and ground to powder with a pre-chilled mortar and pestle. Precisely weighed 50 mg of finely powdered plant material was transferred into glass vials, whereupon metabolite analysis was subsequently conducted using silicone tubing (ST) in accordance with established methodologies [66,67]. ST segments measuring 5 mm in length (1 mm i.d. × 1.8 mm o.d.) were prepared. Each vial received 1 mL of saturated CaCl_2_ solution to suppress enzymatic activity, supplemented with nonyl acetate (10 ng/mL) as an internal standard. A single ST piece was added to each vial, which was then sealed and incubated for a minimum of 8 h at room temperature with shaking at 600 rpm. Following incubation, ST segments were retrieved, rinsed with distilled water, and dried under a gentle nitrogen gas stream. Dried ST segments were placed into 85 mm thermal desorption (TD) tubes for analysis.

Metabolite profiling was conducted using a TD system (TD-20) coupled to a gas chromatography-mass spectrometry (GC-MS) instrument equipped with a wax column (30 m × 0.25 mm × 0.25 μm). TD was performed for 15 min with nitrogen flow at 100 mL/min to eliminate residual water and contaminants. The GC temperature program started at 40 °C for 5 min, increased at 5 °C/min to 115 °C, then ramped at 30 °C/min to 230 °C for column cleaning. MS data acquisition was terminated at 18 min after compound elution to avoid contamination during the cleaning phase. Peak areas were integrated and normalized to fresh tissue mass and the internal standard. For absolute quantification, calibration curves were constructed using serial dilutions of authentic reference standards prepared in an identical CaCl_2_ solution supplemented with nonyl acetate. These standards were processed with ST following the identical procedure as the samples. Quantification was based on calibration responses relative to the internal standard [68,69].

### 4.9. Determination of Antioxidant Enzyme Activities

Fresh leaf and root tissues (0.5 g each) were homogenized in 50 mM in ice-cold potassium phosphate buffer (PBS; pH 7.8), followed by a 15 min centrifugation at 10,000 rpm at 4 °C to separate the supernatant. The supernatant containing soluble enzymes was immediately utilized for enzymatic assays. SOD activity was assayed as the inhibition of photochemical reduction of NBT [70]. CAT activity was quantified by following the disappearance of H_2_O_2_ [71]. APX activity was determined by measuring the rate of ascorbate oxidation [72], and GR activity was evaluated as described in [64].

### 4.10. Relative Gene Expression Analysis by Quantitative qRT-PCR

The RNA extraction kit (Takara, Kusatsu, Japan) was used on 0.1 g samples from leaf and root tissues to obtain total RNA, which was then treated with gDNA Eraser to remove genomic DNA contamination. First-strand cDNA was synthesized from 1 μg of total RNA using the PrimeScript^TM^ RT reagent kit (Takara, Kusatsu, Japan), and qRT-PCR was performed in triplicate with the SYBR^®^ Premix Ex Taq II (Takara, Kusatsu, Japan) on a CFX96 Real-Time PCR Detection System (Bio-Rad, Hercules, CA, USA). Relative gene expression levels were analyzed using the 2^−ΔΔCT^ method [73], and primer sequences were listed in Table S2.

### 4.11. Statistical Analysis

Data were analyzed using one-way analysis of variance (ANOVA) separately for each cultivar, with treatment as the main factor, using Statistix (version 8.1; Statistix 8.1). Results are presented as mean ± standard deviation (SD; *n* = 3). Mean comparisons were performed using Tukey’s test, and different lowercase letters indicate significant differences among treatments at *p* ≤ 0.05. Graphs were generated using GraphPad Prism (version 10.5.0) software. PCA was performed on 18 physiological traits encompassing growth parameters (leaf and root fresh/dry weights, shoot and root lengths) and photosynthetic attributes (Chl a and b, Pn, gs, Tr, F_v_/F_m_). Additionally, oxidative stress markers, including MDA, H_2_O_2_, and O_2_^•−^, were recorded in both leaves and roots. Data were centered and scaled prior to analysis using the prcomp function in R. PCA score plots with 95% confidence ellipses, and biplots were generated to visualize cultivar and treatment separations.

Volatile metabolite profiles (14 metabolites for Zheda 635; 22 for Zheda 622) were log_2_-transformed [log_2_(*x* + 1)] and subjected to hierarchical clustering using Ward’s method (ward.D2) with row-scaling (z-score normalization). Heatmaps were visualized using the *pheatmap* package with the RdYlBu color palette. PCA biplots were constructed for metabolite data, with the top five contributing metabolites displayed as loading vectors. For individual metabolite comparisons, one-way ANOVA followed by Tukey’s HSD test (α = 0.05) was performed on data from seven biological replicates per treatment. Compact letter displays were generated using the multcompView package. All analyses were conducted in R using the ggplot2, cowplot, ggrepel, and tidyverse packages.

## 5. Conclusions

This comprehensive study demonstrates that SeNPs effectively mitigate Cu toxicity in *B. napus* through multifaceted protective mechanisms. SeNPs successfully alleviated Cu-induced growth inhibition, restored photosynthetic efficiency, and enhanced antioxidant defense systems across multiple cultivars. The protective effects operate through reduced Cu uptake, enhanced enzymatic antioxidant activities, and maintenance of cellular redox homeostasis, which minimizes oxidative damage. Significant cultivar-specific responses revealed substantial genetic variation in Cu tolerance mechanisms within *B. napus*. Zheda 635 demonstrated superior tolerance through metabolic stability and efficient primary defenses, while Zheda 622 exhibited greater sensitivity, characterized by extensive metabolic reprogramming and higher oxidative stress. The differential accumulation of VOCs and distinct PCA clustering patterns reflected these contrasting metabolic strategies in response to stress conditions. These findings establish that SeNPs represent a viable nanomaterial option for eco-friendly soil remediation, addressing both crop productivity and food safety concerns. Future investigations should prioritize elucidating molecular mechanisms of Cu-Se interactions, evaluating long-term effects on soil health and food safety.

## Data Availability

The original contributions presented in this study are included in the article/Supplementary Material. Further inquiries can be directed to the corresponding authors.

## Supplementary Materials

The following supporting information can be downloaded at: https://www.mdpi.com/article/10.3390/plants15091333/s1. Figure S1: Phenotypic responses of *B. napus*: (A) Zheda 635, the tolerant genotype; (B) Zheda 622, the sensitive genotype under different treatment conditions.; Figure S2. Physicochemical characterization of selenium nanoparticles (SeNPs) showing (A) FTIR spectrum and (B) XRD pattern.; Figure S3: Synergistic effects of copper (0, 100 and 200 μM), and exogenous SeNPs (10 and 25 μM) and their interactions on growth parameters in *B. napus* cultivars (Zheda 635, Zheda 630, ZS 758 and Zheda 622): (A) leaf fresh weight (LFW), (B) leaf dry weight (LDW), (C) root fresh weight (RFW), (D) root dry weight (RDW), (E) shoot length (SL), and (F) root length (RL). Bars indicate mean ± SD. Different letters show significant differences at *p* ≤ 0.05 (Tukey’s test).; Figure S4: Synergistic effects of copper (0, 100, and 200 μM) and exogenous SeNPs (10 and 25 μM) and their interactions on nutrient contents in leaf and root of *B. napus* cultivars in Zheda 635 (most tolerant) and Zheda 622 (most sensitive): (A) Fe in leaf, (B) Fe in root, (C) P in leaf, (D) P in root, (E) K in leaf, and (F) K in root. Bars indicate mean ± SD. Different letters show significant differences at *p* ≤ 0.05 (Tukey’s test).; Table S1: Treatments are shown in the completely randomized design.; Table S2: Oligonucleotide primer sequences used for gene expression (qRT-PCR) of antioxidants analysis. Table S3. Eigenvalues, proportion of variance, and cumulative variance explained by the first six principal components derived from 18 physiological traits measured across four *Brassica napus* cultivars (622, 630, 635, 758) under nine SeNPs and Cu stress treatments (*n* = 36). Table S4. Eigenvector loadings, percentage contributions, and squared cosine values (cos^2^) for each physiological trait on PC1 and PC2.

## Abbreviations

The following abbreviations are used in this manuscript:

SeNPs	Selenium nanoparticles
Cu	Copper
*B. napus*	*Brassica napus*
EDS	Energy-dispersive X-ray spectroscopy
FTIR	Fourier-transform infrared spectroscopy
XRD	X-ray diffraction
SEM	Scanning electron microscopy
TEM	Transmission electron microscopy
PCA	Principal component analysis
qRT-PCR	Quantitative real-time polymerase chain reaction
ICP-OES	Inductively coupled plasma optical emission spectrometry
GC-MS	Gas chromatography-mass spectrometry
PSII	Photosystem II
Fv/Fm	Maximum quantum efficiency of photosystem II
NBT	Nitroblue tetrazolium
MDA	Malondialdehyde
H_2_O_2_	Hydrogen peroxide
O_2_^•–^	Superoxide radical
VOC	Volatile organic compound
HNS	Hoagland nutrient solution
SOD	Superoxide dismutase
CAT	Catalase
APX	Ascorbate peroxidase
GR	Glutathione reductase
