# Supplementary material for: Selenium Nanobiostimulants Attenuate Copper-Induced Oxidative Damage in Brassica napus Through Genotype-Specific Antioxidant and Metabolic Adaptation
Source: Plants (Basel). 2026 Apr 27;15(9):1333. doi: 10.3390/plants15091333 (PMC13164830; doi:10.3390/plants15091333)
Supplement: Supplementary file 1 [file plants-15-01333-s001.zip › plants-4275016-supplementary.pdf]

Supplementary material

Selenium Nanobiostimulants Attenuate Copper-Induced Oxidative Damage in *Brassica napus* Through Genotype-Specific Antioxidant and Metabolic Adaptation

Sundas Fatima [a, 1], Muhammad Arslan Yousaf [a, 1], Saba Yaseen [a], Muhammad Kamran [a], Basharat Ali [b], Yingying Zhou [a], Asad Ullah [a], Fangbin Cao [a], Skhawat Ali [a, *], Weijun Zhou [a, *]

[a] *Institute of Crop Science, Zhejiang Key Laboratory of Crop Germplasm Innovation and Utilization, Zhejiang University, Hangzhou 310058, China.*

[b] *Department of Agricultural Engineering, Khwaja Fareed University of Engineering and Information Technology, Rahim Yar Khan 64200, Pakistan.*

[1] *These author contributed equally.*

[*] *Corresponding author email: skhawataligill@yahoo.com* ***(SA)***; *wjzhou@zju.edu.cn* **(WZ)**

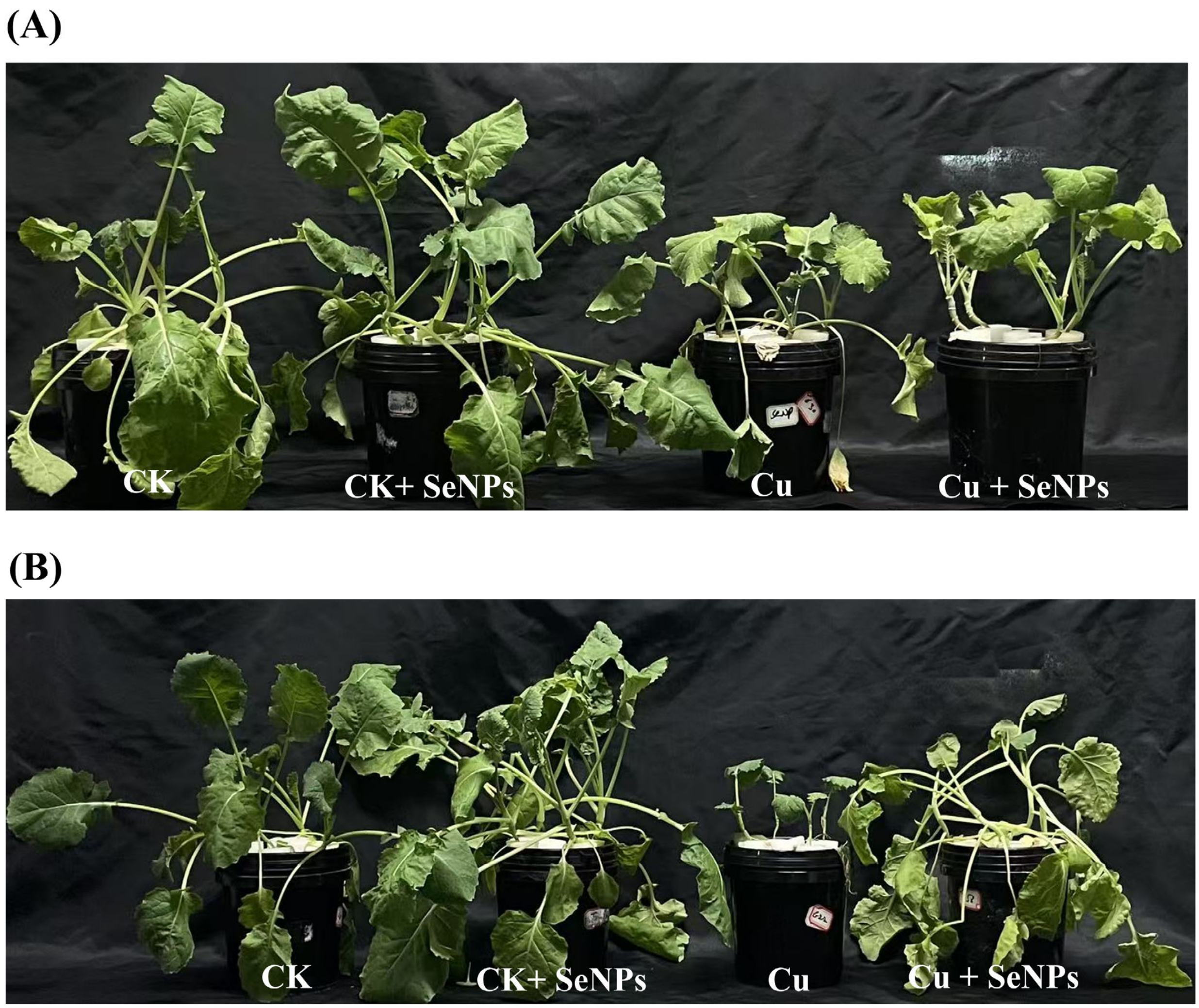

Figure S1. Phenotypic responses of *B. napus*: (A) Zheda 635, the tolerant genotype; (B) Zheda 622, the sensitive genotype under different treatment conditions.

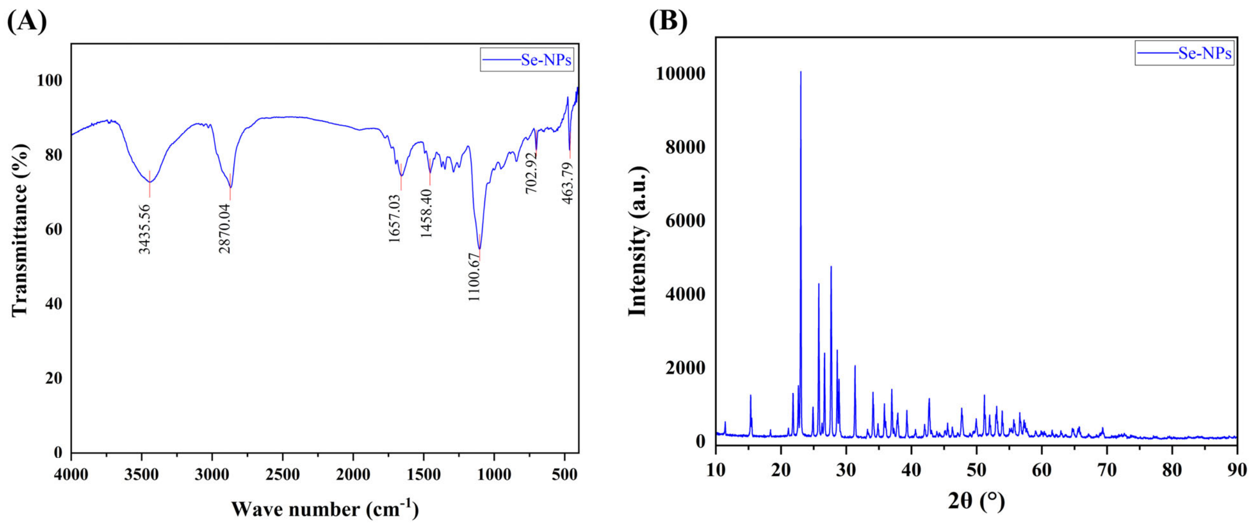

Figure S2. Physicochemical characterization of selenium nanoparticles (SeNPs) showing (A) FTIR spectrum and (B) XRD pattern

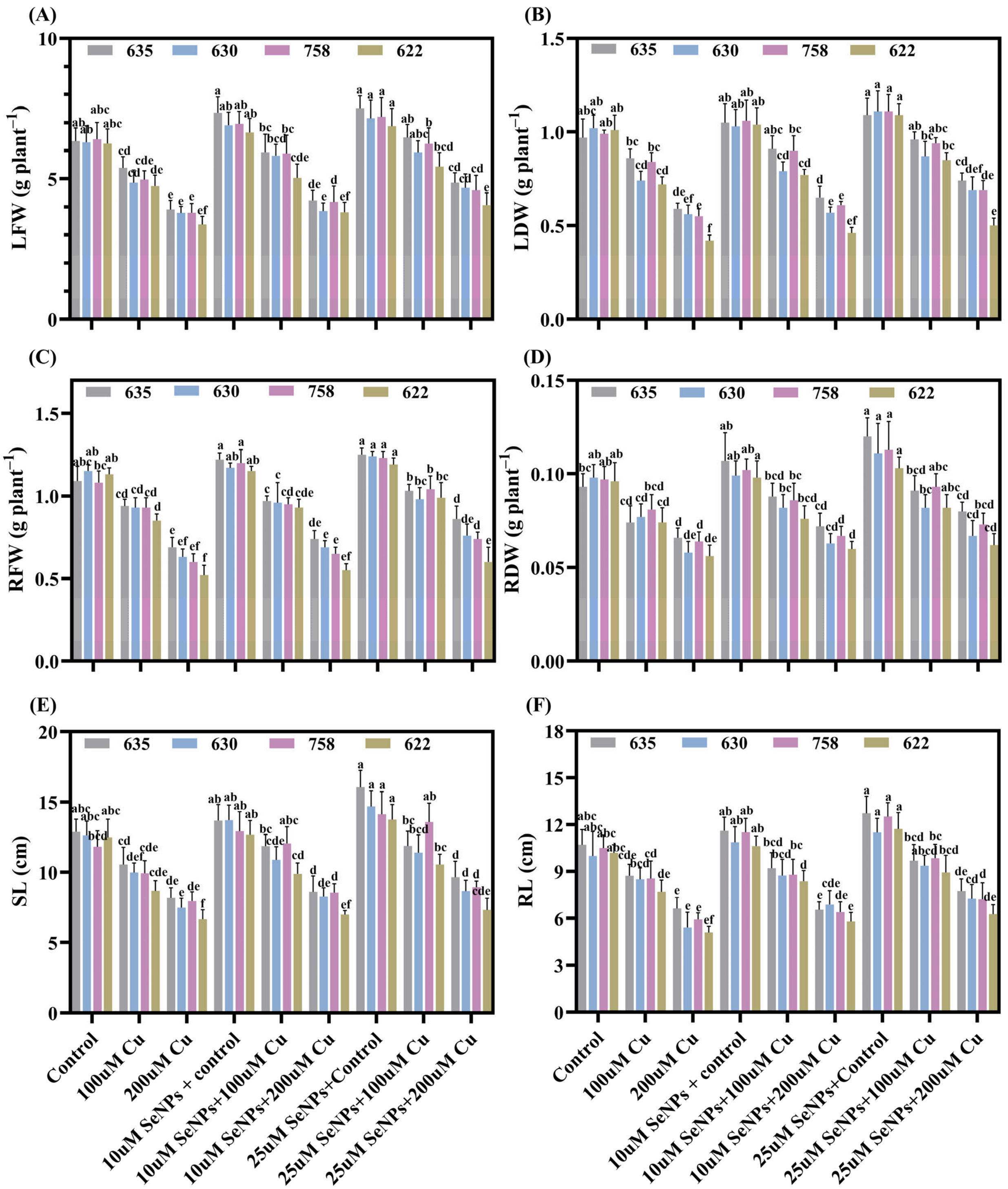

Figure S3. Synergistic effects of copper (0, 100 and 200 µM), and exogenous SeNPs (10 and 25 µM) and their interactions on growth parameters in *B. napus* cultivars (Zheda 635, Zheda 630, ZS 758 and Zheda 622): (A) leaf fresh weight (LFW), (B) leaf dry weight (LDW), (C) root fresh weight (RFW), (D) root dry weight (RDW), (E) shoot length (SL) and (F) root length (RL). Bars are presented as mean ± SD (n = 3). Bars sharing the same lowercase letter are not significantly different, whereas bars with different letters differ significantly according to Tukey's HSD test at $p \leq 0.05$, with comparisons made within each cultivar.

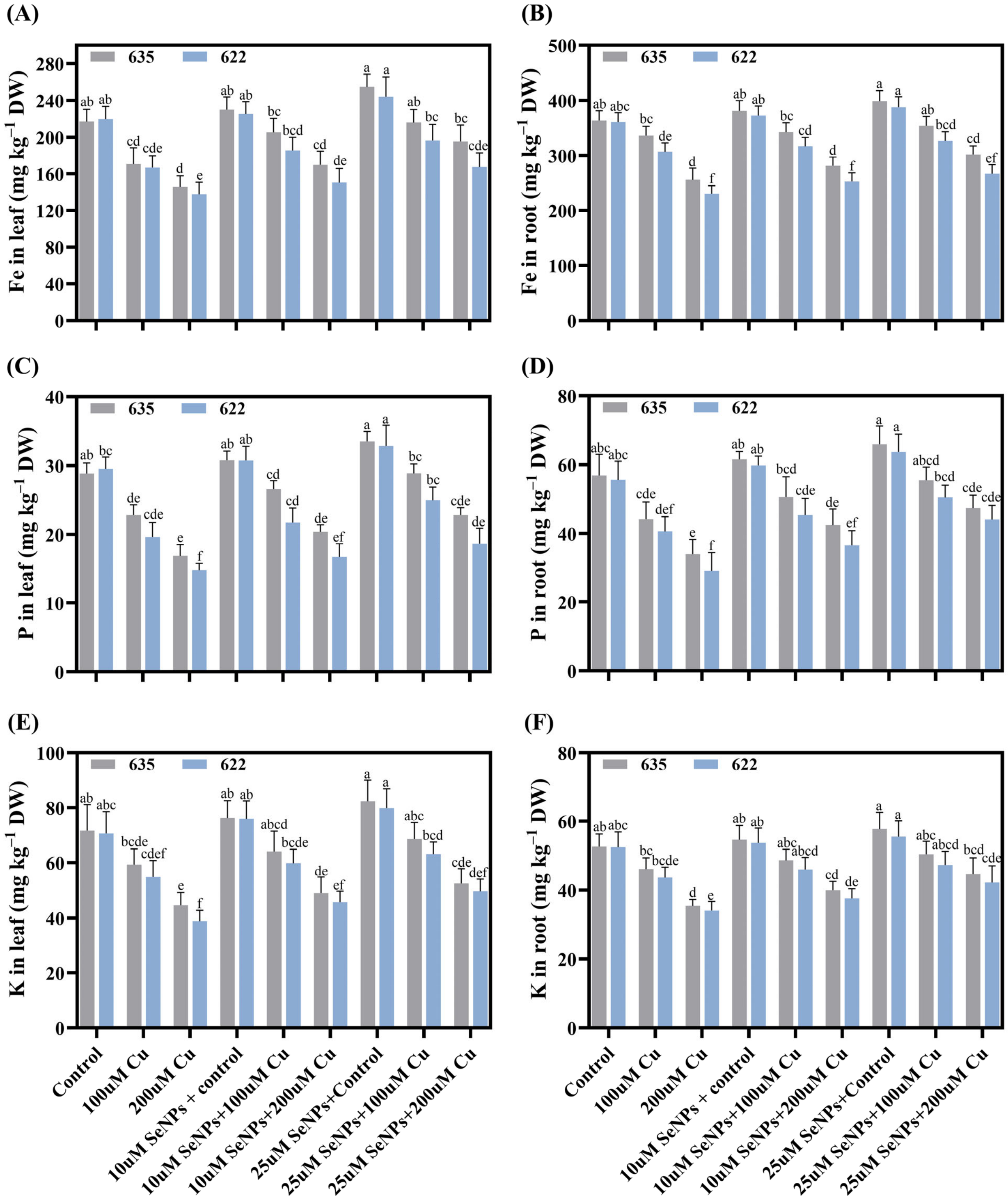

Figure S4. Synergistic effects of copper (0, 100 and 200 μM) and exogenous SeNPs (10 and 25 μM) and their interactions on nutrient contents in leaf and root of *B. napus* cultivars in Zheda 635 (most tolerant) and Zheda 622 (most sensitive): (A) Fe in leaf, (B) Fe in root, (C) P in leaf, (D) P in root, (E) K in leaf, and (F) K in root. Bars are presented as mean ± SD (n = 3). Bars sharing the same lowercase letter are not significantly different, whereas bars with different letters differ significantly according to Tukey's HSD test at $p \leq 0.05$, with comparisons made within each cultivar.

Table S1. Treatments are shown in the completely randomized design.

Treatment No.	Description
T0	Control (CK)
T1	100 μM Cu (alone)
T2	200 μM Cu (alone)
T3	10μM SeNPs + control
T4	10μM SeNPs + 100μM Cu
T5	10μM SeNPs + 200μM Cu
T6	25μM SeNPs + Control
T7	25μM SeNPs + 100μM Cu
T8	25μM SeNPs + 200μM Cu

Table S2. Oligonucleotide primer sequences used for gene expression (qRT-PCR) of antioxidants analysis

Gene name	Forward	Reverse
BnaSOD	ACGGTGTGACCACTGTGACT	GCACCGTGTTGTTTACCATC
BnaCAT	TCGCCATGCTGAGAAGTATC	TCTCCAGGCTCCTTGAAGTT
BnaAPX	ATGAGGTTTGACGGTGAGC	CAGCATGGGAGATGGTAGG
BnaGR	AAGCTGGAGCTGTGAAGGTT	AGACAGTGTTCGCAAAGCAG
Actin	TTGGGATGGACCAGAAGG	TCAGGAGCAATACGGAGC

Table S3. Eigenvalues, proportion of variance, and cumulative variance explained by the first six principal components derived from 18 physiological traits measured across four *Brassica napus* cultivars (622, 630, 635, 758) under nine SeNPs and Cu stress treatments (n = 36). Data were centered and scaled prior to PCA.

PC	Eigenvalue	Variance (%)	Cumulative (%)	Interpretation
**PC1**	10.875	58.7	58.7	Growth vs. stress axis
**PC2**	6.201	33.5	92.2	ROS and root trait axis
**PC3**	0.774	4.2	96.4	Minor residual variation
**PC4**	0.357	1.9	98.3	Minor residual variation
**PC5**	0.146	0.8	99.1	Negligible
**PC6**	0.057	0.3	99.4	Negligible

Table S4. Eigenvector loadings, percentage contributions, and squared cosine values (cos^2) for each physiological trait on PC1 and PC2. Traits are sorted by PC1 loading (descending). Contributions were calculated as ($loading^2$ / $\Sigma loadings^2$) × 100.

Trait	PC1 Loading	PC1 Contrib. (%)	PC2 Loading	PC2 Contrib. (%)	Cos^2 (PC1)	Cos^2 (PC2)
**LDW**	+0.3009	9.05	-0.0144	0.02	0.091	0.000
**RL**	+0.3006	9.04	-0.0045	0.00	0.090	0.000
**SL**	+0.3003	9.02	-0.0004	0.00	0.090	0.000
**LFW**	+0.3001	9.00	-0.0145	0.02	0.090	0.000
**Pn**	+0.2995	8.97	-0.0547	0.30	0.090	0.003
**Gs**	+0.2287	5.23	-0.2592	6.72	0.052	0.067
**Chl b**	+0.2171	4.71	-0.2702	7.30	0.047	0.073
**H_2O_2 root**	-0.0240	0.06	+0.4034	16.27	0.001	0.163
**H_2O_2 leaf**	-0.0299	0.09	+0.4018	16.15	0.001	0.161
**RFW**	-0.1508	2.28	-0.3178	10.10	0.023	0.101
**RDW**	-0.1621	2.63	-0.3032	9.20	0.026	0.092
**Chl a**	-0.1969	3.88	-0.2838	8.05	0.039	0.081
**MDA root**	-0.2068	4.27	+0.2938	8.63	0.043	0.086
**Tr**	-0.2160	4.66	-0.2556	6.53	0.047	0.065
**Fv/Fm**	-0.2282	5.21	-0.2393	5.73	0.052	0.057
**MDA leaf**	-0.2401	5.77	+0.2041	4.16	0.058	0.042
**$O_2^{\bullet-}$ root**	-0.2789	7.78	-0.0831	0.69	0.078	0.007
**$O_2^{\bullet-}$ leaf**	-0.2890	8.35	+0.0347	0.12	0.084	0.001
